# Selfish mutations dysregulating RAS-MAPK signaling are pervasive in aged human testes

**DOI:** 10.1101/gr.239186.118

**Published:** 2018-12

**Authors:** Geoffrey J. Maher, Hannah K. Ralph, Zhihao Ding, Nils Koelling, Hana Mlcochova, Eleni Giannoulatou, Pawan Dhami, Dirk S. Paul, Stefan H. Stricker, Stephan Beck, Gilean McVean, Andrew O.M. Wilkie, Anne Goriely

**Affiliations:** 1 Clinical Genetics Group, MRC-Weatherall Institute of Molecular Medicine, University of Oxford, Oxford OX3 9DS, United Kingdom;; 2 Nuffield Division of Clinical Laboratory Sciences, Radcliffe Department of Medicine, University of Oxford, Oxford OX3 9DS, United Kingdom;; 3 Medical Genomics, UCL Cancer Institute, University College London, London WC1E 6BT, United Kingdom;; 4 Big Data Institute, Li Ka Shing Centre for Health Information and Discovery, University of Oxford, Oxford OX3 7LF, United Kingdom

## Abstract

Mosaic mutations present in the germline have important implications for reproductive risk and disease transmission. We previously demonstrated a phenomenon occurring in the male germline, whereby specific mutations arising spontaneously in stem cells (spermatogonia) lead to clonal expansion, resulting in elevated mutation levels in sperm over time. This process, termed “selfish spermatogonial selection,” explains the high spontaneous birth prevalence and strong paternal age-effect of disorders such as achondroplasia and Apert, Noonan and Costello syndromes, with direct experimental evidence currently available for specific positions of six genes (*FGFR2*, *FGFR3*, *RET*, *PTPN11*, *HRAS*, and *KRAS*). We present a discovery screen to identify novel mutations and genes showing evidence of positive selection in the male germline, by performing massively parallel simplex PCR using RainDance technology to interrogate mutational hotspots in 67 genes (51.5 kb in total) in 276 biopsies of testes from five men (median age, 83 yr). Following ultradeep sequencing (about 16,000×), development of a low-frequency variant prioritization strategy, and targeted validation, we identified 61 distinct variants present at frequencies as low as 0.06%, including 54 variants not previously directly associated with selfish selection. The majority (80%) of variants identified have previously been implicated in developmental disorders and/or oncogenesis and include mutations in six newly associated genes (*BRAF*, *CBL*, *MAP2K1*, *MAP2K2*, *RAF1*, and *SOS1*), all of which encode components of the RAS-MAPK pathway and activate signaling. Our findings extend the link between mutations dysregulating the RAS-MAPK pathway and selfish selection, and show that the aging male germline is a repository for such deleterious mutations.

The timing, location, and functional effects of spontaneous mutations determine the distribution and phenotypes of mutant cells within the body. This can have a variety of impacts on the health of an individual and, potentially, their offspring. Spontaneous mutations occurring during early post-zygotic development lead to widespread tissue mosaicism that, depending on context, may be phenotypically undetectable or cause so-called “somatic” disorders (Campbell et al. 2015). Such early post-zygotic mosaicism occurs commonly, with up to 22% of apparently de novo point mutations (DNMs) detectable in a child's blood sample likely to have occurred after fertilization (Acuna-Hidalgo et al. 2015; Krupp et al. 2017). A corollary is that a further ∼4%–10% of DNMs and ∼4% of copy-number variants (CNVs) present in a child can be detected at a low level in one of the parent's somatic (usually blood or saliva) samples and are therefore in fact inherited; as these would have occurred early during parental post-zygotic development (before the separation of the somatic and gonadal lineages), they are associated with a significant risk of recurrence (Campbell et al. 2014; Acuna-Hidalgo et al. 2015; Rahbari et al. 2016; Krupp et al. 2017). In contrast, spontaneous mutations occurring post-natally contribute to tissue-specific low-level mosaicism, the formation of benign tumors, or cancer, depending on the functional consequence(s) of the acquired mutation(s), the clonal dynamics of the tissue involved, and the state of the niche (Klein et al. 2010a; Vermeulen et al. 2013; Holstege et al. 2014; Swanton 2015). This latter phenomenon has been documented in apparently healthy somatic tissues that display stem cell replacement (e.g., skin, colon, small intestine, and blood), where low levels (∼1%–10%) of clonal mutations are prevalent and their incidence and frequency increase with age (Hafner et al. 2010; Laurie et al. 2012; Genovese et al. 2014; Jaiswal et al. 2014; Martincorena et al. 2015, 2017; McKerrell et al. 2015; Acuna-Hidalgo et al. 2017; Coombs et al. 2017; Zink et al. 2017).

Analogous to the post-natal occurrence of somatic mutations, we previously demonstrated a similar phenomenon, termed selfish spermatogonial selection, that occurs in the testes of adult men as they age. However, because the testis contains germ cells that, upon fertilization, will carry the genetic information across generations, this process has important reproductive implications, being associated with an increased prevalence of pathogenic DNMs in the next generation. Despite the relatively low average human germline point mutation rate of ∼1.2 × 10^−8^ per nucleotide per generation (Kong et al. 2012; Goldmann et al. 2016; Jonsson et al. 2017), specific “selfish” DNMs in *FGFR2*, *FGFR3*, *HRAS*, *PTPN11*, and *RET* are observed up to 1000-fold more frequently in offspring (Goriely and Wilkie 2012). These pathogenic mutations, which cause developmental disorders that show an extreme paternal bias in origin and an epidemiological paternal age-effect (collectively referred to as PAE disorders; e.g., achondroplasia; Apert, Costello, and Noonan syndromes; multiple endocrine neoplasia type 2a/b), are identical (or allelic) to oncogenic driver mutations in tumors (Goriely and Wilkie 2012). We have proposed that although they arise at the normal background rate in male germline stem cells (spermatogonia), selfish mutations alter the behavior of spermatogonia within the testis. In a process akin to oncogenesis, these gain-of-function mutations provide a selective advantage that may involve increasing the rate of symmetrical divisions of the mutant spermatogonia (Qin et al. 2007; Choi et al. 2008, 2012; Giannoulatou et al. 2013; Yoon et al. 2013; Martin et al. 2014), leading to their clonal expansion over time, which results in increased apparent mutation levels in sperm with age (Goriely and Wilkie 2012; Maher et al. 2014).

Three methods have previously been used to detect selfish mutations in the male germline, each of which has been limited in their ability to evaluate the process at scale: (1) quantification in sperm, (2) quantification in testis biopsies, and (3) direct identification in seminiferous tubules. Detecting selfish mutations in sperm, in which individual mutations are present at levels ranging from 10^−3^ to <10^−6^, requires ultrasensitive techniques that have limited reliable quantitative analysis to small regions of 1–6 nucleotides across five locations in *FGFR2* (×2) (Goriely et al. 2003, 2005; Yoon et al. 2009), *FGFR3* (×2) (Tiemann-Boege et al. 2002; Goriely et al. 2009), and *HRAS* (Supplemental Table S1; Giannoulatou et al. 2013). To circumvent the technical challenges caused by mutational dilution within an entire ejaculate, mutations may alternatively be identified following systematic dissection and sequencing of DNA extracted from discrete testicular biopsies. Germ cells (from diploid spermatogonia to haploid spermatozoa) are located in long (up to ∼80 cm) highly convoluted and tightly packed seminiferous tubules, comprising approximately 300–500 per testis (Glass 2005). As clonally expanding mutant spermatogonia are physically restricted to the tubules in which they arise, their geographical distribution within the testis is confined to specific regions: The existence of such localized foci has been demonstrated for selfish mutations in four genes (*FGFR2*, *FGFR3*, *PTPN11*, *RET*) (Qin et al. 2007; Choi et al. 2008, 2012; Dakouane Giudicelli et al. 2008; Shinde et al. 2013; Yoon et al. 2013; Eboreime et al. 2016). Finally, mutant clones have been directly visualized in sections of formalin-fixed paraffin-embedded (FFPE) normal human testes using immunohistochemical approaches to reveal abnormal expression of spermatogonial antigens (Lim et al. 2012; Maher et al. 2016a). Microdissection of tubules exhibiting enhanced antigen staining and subsequent whole-genome amplification facilitated the screening of over 100 genes, identifying nine new selfish mutations, including one in a novel gene (*KRAS*) (Supplemental Table S1). However, this approach is limited both by the need to source fixed testis samples with good tissue morphology and DNA preservation and by the high threshold required for successful immunohistochemical detection (Maher et al. 2016a,b).

Owing to the limitations outlined above, experimental evidence of clonal expansion has so far been restricted to activating mutations at 16 codons in only six genes (Supplemental Table S1), all encoding members of the receptor tyrosine kinase (RTK)-RAS-MAPK signaling pathway. Here, we hypothesized that other variants dysregulating the RAS-MAPK pathway, and/or other pathways controlling spermatogonial stem cell homeostasis, may be under positive selection in the male germline (Goriely and Wilkie 2012; Goriely et al. 2013). To reduce the required assay sensitivity compared with bulk semen analysis, and hence substantially widen the extent of the genomic target that could feasibly be analyzed in a single experiment, we exploited approach 2 above. By combining systematic dissection of testicular biopsies with massively parallel simplex PCR and ultradeep sequencing of mutational hotspots in 67 genes, we present the most comprehensive survey of mutations clonally enriched in the human testis to date.

## Results

To perform a discovery screen to identify novel mutations and genes under selection in the male germline, we systematically biopsied human testes (with no known phenotypic indicators) following the experimental design summarized in Supplemental Figure S1. A total of 276 small biopsies (∼60–180 mm^3^) from five men (age range, 34–90 yr; median, 83 yr) were screened by ultradeep Illumina sequencing (about 16,000× post-filtering) of a panel of candidate loci (corresponding to 59.4 kb of targeted genomic sequence across 500 amplicons, covering mutational hotspots in 61 candidate genes and genomic regions of 10 negative control genes [neutral-test]; see Methods for criteria used to include loci in screen), amplified using massively parallel simplex PCR (RainDance Thunderstorm). To detect low-level mosaicism in individual biopsies (∼0.1%–3.0%), the background at each genomic location was independently estimated for all 431 (of 500) amplicons (in 67 of 71 genes) that passed quality control (QC) (Supplemental Table S2). After normalization, a statistical model was applied to call outlier nonconsensus variants at each genomic position (within each amplicon): A minimum threshold of 10 variant reads and median coverage of greater than 5000× was implemented to reduce false-positive calls. As a conservative prioritization strategy, only variants with two or more independent calls were further studied, resulting in a set of 374 variant calls located at 361 genomic locations (see Methods). Visualization and manual curation of each of these calls identified 115 higher-confidence candidate variants, distributed at 105 genomic positions across 165 biopsies (all 115 variants are detailed in Supplemental Table S3).

As calling variants at low levels (<1%) is subject to PCR artifacts and sequencing errors (Minoche et al. 2011; Hestand et al. 2016; Salk et al. 2018), we developed a tiered strategy for further variant prioritization. We reasoned that variants called independently in overlapping amplicons or in sample replicates (12 biopsies were amplified and sequenced in duplicate) were least likely to be artifactual (Tier 1 variants, Table 1). Eighteen of the 40 Tier 1 variants (with VAF ranging from 0.10% to 2.63%) were rescreened by PCR or by using single-molecule molecular inversion probes (smMIPs) and ultradeep MiSeq sequencing (about 30,000×). Seventeen of the 18 (94%) variants were validated, suggesting the great majority of Tier 1 variants are true-positive calls (Table 1; Supplemental Table S3). Among the Tier 1 variants are five mutations previously associated experimentally with selfish selection: *FGFR2* c.755C > G (p.Ser252Trp – Apert syndrome), c.758C > G (p.Pro253Arg – Apert syndrome) and c.870G > T (p.Trp290Cys – Pfeiffer syndrome), *KRAS* c.182A > G (p.Gln61Arg – oncogenic), and *PTPN11* c.215C > T (p.Ala72Val – oncogenic) (Table 1). This strong enrichment for canonical examples of selfish mutations (Supplemental Table S1) provided initial validation of our experimental approach and starting hypothesis.

**Table 1.**
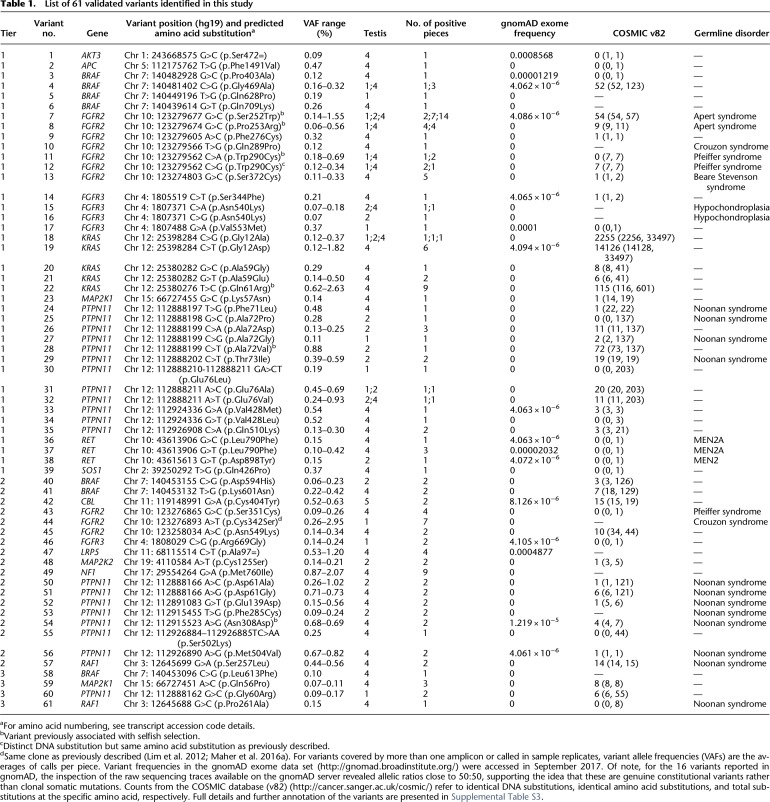
List of 61 validated variants identified in this study

Within the panel, the majority (88.7%) of callable (i.e., excluding primer sequences and amplicons with low QC) regions were represented by a single amplicon, and only 12 biopsies were sequenced in duplicate (Supplemental Table S4): Hence, we next investigated variants that were called in single amplicons in two or more biopsies, at VAF of ≥0.2% in at least one biopsy (Tier 2). Twenty-six Tier 2 variants were identified and were rescreened by direct PCR amplification or smMIPs and ultradeep MiSeq sequencing, 18 (69%) of which were true positives (Table 1; Supplemental Table S3). Notably, all (14/14) of the known pathogenic variants were validated, but only four of the 12 variants without prior disease association were true positives. In biopsy 4D25, *PTPN11* c.1504T > A (p.Ser502Thr – Noonan syndrome) was called as a single-nucleotide variant, but on validation, it was identified as a double-nucleotide substitution c.1504_1505delTCinsAA (p.Ser502Lys). Next, 29 variants with a VAF of 0.1%–0.2% called in a single amplicon in two or more biopsies (Tier 3) were identified. Only four of the 22 (18%) resequenced Tier 3 variants were validated, suggesting that in this lower frequency range, the majority of calls are artifactual (Table 1; Supplemental Table S3). Owing to the low validation rate of variants with VAFs of 0.1%–0.2%, none of the remaining 20 calls that exhibited VAF < 0.1% (Tier 4) variants were rescreened for validation (Supplemental Table S3).

**Figure 1.**
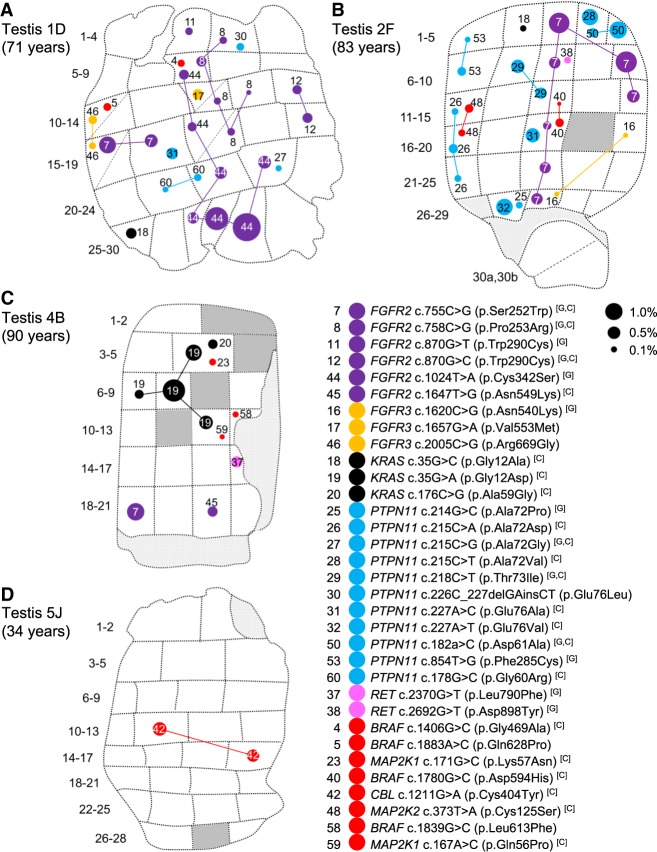
Distribution of validated variants in testis slices 1D (*A*), 2F (*B*), 4B (*C*), and 5J (*D*). Testicular biopsy numbers are located to the *left* of each testis slice. Some biopsies were further dissected into two pieces of which the orientation is unknown; these are indicated with a diagonal dashed line (e.g., Tes2F 30a,b). Each variant has a distinct number (as listed in Table 1) and is colored according to gene—*FGFR2* (purple), *FGFR3* (orange), *KRAS* (black), *PTPN11* (blue), *RET* (pink), and newly associated gene (red)—and is also indicated on the figure key. The size of each circle is proportional to the observed variant allele frequency (VAF) in each biopsy as indicated by black dots on the figure key. Identical variants in different biopsies have been connected by lines that likely track the seminiferous tubule trajectory across the testis and therefore may represent a single “clonal event”; note that the path of the clone has been arbitrarily drawn and may not represent the true trajectory of the tubule. Dark gray segments represent biopsies that were not sequenced due to insufficient material quality/quantity (see Methods). Light gray segments represent nontubular regions of tissue. The age of the individual from whom the testis was collected is indicated on the figure (for further details on the testicular samples, see Supplemental Table S5). The remaining five slices of Tes4 are presented in Supplemental Figure S2. Tes3D is omitted as no variants were identified. Variants are numbered in order of tier: Tier 1 (1–39), Tier 2 (40–57), Tier 3 (58–61). Letters in brackets refer to variants associated with germline disorders [G] and/or reported in the COSMIC database [C]; for further details, see also Table 1 and Supplemental Table S3.

**Figure 2.**
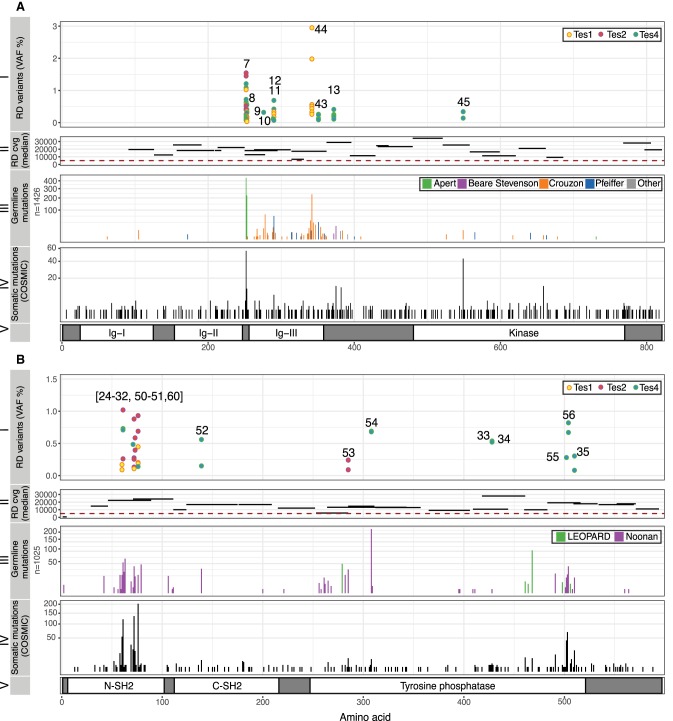
Spontaneous mutations in *FGFR2* (*A*) and *PTPN11* (also known as *SHP2*; *B*) identified in testicular biopsies. (*A*, *I*) Ten validated variants positioned along the amino acid sequence of FGFR2 (*x*-axis, see panel *V*), ranging in VAF from 0.06% to 2.95% (*y*-axis), identified in Tes1D, Tes2F, and Tes4. Numbers correspond to those in Table 1; two different variants (c.870G > C or T) predicted to cause the same p.Trp290Cys substitution (nos. 11, 12) were identified. (*II*) Relative location and length of amplicons used to sequence main hotspots of *FGFR2* are plotted on the *x*-axis. Median coverage per amplicon is plotted on the *y*-axis. All amplicons had median coverage above the cut-off (red dashed line) of 5000×. (*III*) Number of reported constitutional variants encoding amino acid substitutions in FGFR2 associated with developmental disorders (sqrt scale) (updated from Wilkie 2005). (*IV*) Number of reported somatic amino acid substitutions in FGFR2 in cancer (COSMIC v82). (*V*) Protein domains of FGFR2. Annotations and protein structure are based on transcript ID NM_000141 and Uniprot ID P21802 (v2017_01), respectively. (*B*, *I*) Twenty validated variants positioned along the amino acid sequence of SHP2 (*x*-axis, see panel *V*), ranging in VAF from 0.09% to 1.02% (*y*-axis), identified in Tes1D, Tes2F, and Tes4. (*II*) Location and size of amplicons used to sequence main hotspots of *PTPN11* are plotted on the *x*-axis. Median coverage per amplicon is plotted on the *y*-axis. All amplicons except one had median coverage above the cut-off of 5000×. (*III*) Number of reported constitutional variants encoding amino acid substitutions in SHP2 associated with developmental disorders (sqrt scale). (*IV*) Number of reported somatic amino acid substitutions in SHP2 in cancer (COSMIC v82). (*V*) Protein domains of SHP2. Annotations and protein structure are based on transcript ID NM_002834 and Uniprot ID Q06124 (v2017_01), respectively.

Overall, we identified 61 distinct variants that were classified as independently validated in a total of 162 analyzed samples (corresponding to 111 mutation-positive testis biopsies) present in 15 of the 67 genes that passed QC and were analyzed in the experiment. Based on the identification of the same variant in testes sourced from different men, we conclude that at least 72 independent mutational events (clones) could be distinguished across the five testes (Table 1; Fig. 1A–D; Supplemental Figs. S2, S3). Two variants (*FGFR2* c.755C > G (p.Ser252Trp) [no. 7] and *KRAS* c.35G > C (p.Gly12Ala) [no. 18]) occurred in three testes and seven in two testes (Fig. 1; Supplemental Fig. S2). All these variants either are recurrent mutations causative of congenital skeletal disorders or are known hotspots in cancer (COSMIC) that may be associated with lethal or as-yet undescribed congenital disorders (Table 1). Figure 2 details all validated variants for the two genes most highly represented in this list: *FGFR2* and *PTPN11* (15 independent mutational events responsible for 10 distinct variants in *FGFR2* [encoding nine pathogenic protein changes] and 22 independent mutational events of 20 distinct variants in *PTPN11*). Their relative locations on the respective protein products show considerable overlap with mutational hotspots previously associated with developmental disorders and cancer. The corollary is that our observations of these mutations in testes are likely to be relevant to the biological origins of the cognate diseases. Similar plots for 13 other genes with validated variants are presented in Supplemental Figure S3.

Previous studies have reported that selfish mutations show a localized focal distribution in the testis (Qin et al. 2007; Choi et al. 2008, 2012; Dakouane Giudicelli et al. 2008; Lim et al. 2012; Shinde et al. 2013; Yoon et al. 2013; Eboreime et al. 2016), with mutations in adjacent biopsies likely tracking single seminiferous tubules and representing the same clonal event (Maher et al. 2016a). By use of the geographical register of the multiple biopsies, the spatial distribution of each variant across the testicular biopsies was investigated (Fig. 1; Supplemental Fig. S2). For example, in six of 153 biopsies across three slices from Tes4, we identified a *KRAS* c.35G > A (p.Gly12Asp) mutation (no. 19). *KRAS* c.35G > A is one of the most frequently reported substitutions in cancer (more than 14,000 records in COSMIC v82), and post-zygotic *KRAS* c.35G > A mutations have been reported to cause arteriovenous malformations of the brain (Nikolaev et al. 2018) and linear nevus sebaceous syndrome (Wang et al. 2015), but it has never been reported as a constitutional mutation. In slice 4B (slice B of testis 4) (Fig. 1C; Supplemental Figs. S2, S3F), this *KRAS* mutation was detected at VAFs ranging from 0.26% to 1.82% in four adjacent biopsies, suggestive of an expansion of a mutational event tracking along the length of a single seminiferous tubule. The same *KRAS* variant was also detected in two neighboring biopsies from slices 4D and 4E, apparently at a distance from the larger clone in slice 4B (Supplemental Fig. S2); this smaller clone may represent a distinct mutational event having occurred in an independent tubule, but the resolving power of the experiment does not exclude the possibility that this is a large clonal event spreading along the length of a single seminiferous tubule.

Owing to the convoluted packing of the seminiferous tubules, individual testicular biopsies contain segments of multiple individual tubules, and in 43 biopsies, more than one variant was identified (Fig. 1A–D; Supplemental Fig. S2; Supplemental Table S3). Mutations with similar distributions across multiple biopsies may represent clones either within the same tubule or in distinct intermingled tubules running alongside each other. For example, two distinct mutations, *MAP2K2* c.373T > A (p.Cys125Ser) (oncogenic) and *PTPN11* c.215C > A (p.Ala72Asp) (oncogenic), are both found in the adjacent biopsies 2F11 and 2F16 (Fig. 1B), with the latter mutation extending into the neighboring biopsy 2F21. In Tes4, four of the six biopsies positive for the oncogenic *KRAS* c.182A > G (p.Gln61Arg) mutation (4E18, 4E25, 4F27, 4G1) were also positive for a synonymous variant in *LRP5* (c.291C > T (p.Ala97=); no prior disease association) (Supplemental Figs. S2, S4).

In contrast to selfish mutations that occur in adult spermatogonia and are therefore restricted to the seminiferous tubules in which they arise, “classical” post-zygotic mosaic mutations occurring in embryonic primordial germ cells, before the formation of the seminiferous tubules, are expected to have a wider geographical distribution in one or both testes. We found one suggestive example of this, an *NF1* c.2280G > A (p.Met760Ile) variant, which exhibited a pattern of occurrence in Tes4 distinct from all the other identified mutations. The variant was originally called in nine biopsies at relatively high VAF (median, 1.1%; range, 0.9%–2.1%) (Supplemental Fig. S2), and inspection of the mutation frequency in each sample (Supplemental Fig. S5) showed numerous other biopsies in Tes4 with elevated VAFs, compatible with an earlier post-zygotic mosaic event. Unfortunately, no other tissue was available from this individual to test whether the variant was restricted to a single testis and/or to the germline tissue.

**Figure 3.**
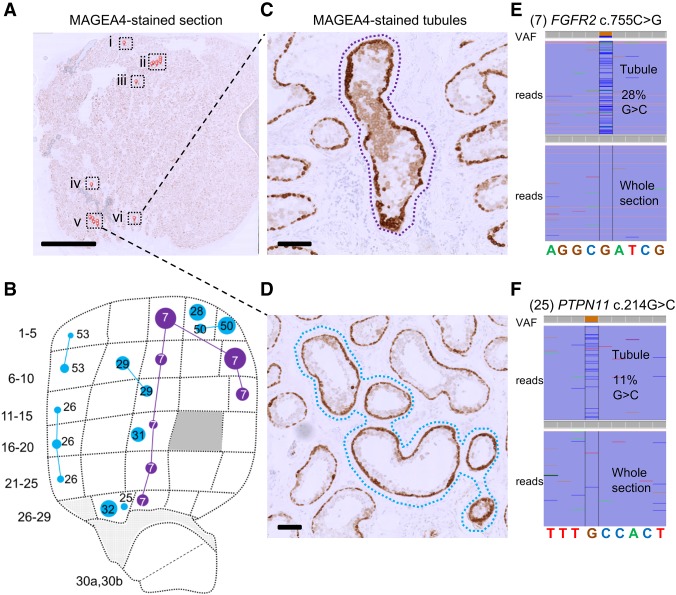
Visualization of mutant tubules in Testis 2. (*A*) A 5-µm-thick section from Tes2E, a FFPE block of tissue adjacent to the testis slice 2F (*B*), immunostained with anti-MAGEA4 antibody to label spermatogonia. Seminiferous tubules with enhanced MAGEA4 immunopositivity, suggestive of the presence of mutant clones are labeled with small red pins and boxed. Scale bar, 5 mm. (*C*,*D*) High-magnification view of cross-sections with MAGEA4-enhanced immunopositivity in two localized areas are labeled with dotted lassoes representing the laser-microdissected regions. Scale bars, 100 µm. (*E*,*F*) Results from targeted resequencing of the microdissected seminiferous tubules labeled by dotted lassoes in *C* and *D*, respectively, viewed in integrated genome viewer (IGV), with local genomic sequence context indicated at the bottom. VAF of mutant reads is indicated on the *top* using color specific for each base pair; spontaneous pathogenic *FGFR2* c.755C > G (no. 7; *E*) and *PTPN11* c.214G > C (no. 25; *F*) variants were identified in DNA extracted from microdissected tubule cross-sections, but not in DNA from the whole-tissue section. Comparison of the MAGEA4 section (*A*) with adjacent testis slice 2F from the RainDance screen (*B*; the same image as in Figure 1B but showing only the targeted *FGFR2* and *PTPN11* mutations) shows that both variants match to a mutation previously identified in the corresponding position of testis slice 2F.

To explore the relationship between mutational events identified using RainDance technology (which inherently involves destruction of the tissue structure of the testis) and the occurrence of mutations in individual seminiferous tubules, we exploited the availability of adjacent FFPE material for two of the testes. In Tes1D, our deep-screening strategy identified a *FGFR2* c.1024T > A (p.Cys342Ser) variant at VAFs ranging from 0.26% to 2.95% in seven contiguous biopsies, suggestive of a clonal event tracking a single seminiferous tubule across the testis (Figs. 1D and 2, variant 44). For this testis, we had previously studied the adjacent FFPE tissue block (Tes1-1 described by Lim et al. 2012; Maher et al. 2016a) using immunohistochemical staining for markers of selfish clones (enhanced MAGEA4 and pAKT immunostaining), followed by laser capture microdissection and targeted resequencing. We previously identified and validated the identical *FGFR2* variant, suggesting that this large mutant clone is present within a significant portion of a single seminiferous tubule that tracks across adjacent testis slices (Maher et al. 2016a). To seek further examples, we undertook a new analysis of putative mutant clones within Tes2E, a FFPE tissue block adjacent to the Tes2F slice, to identify individual tubular cross-sections exhibiting enhanced MAGEA4 immunostaining (Fig. 3A); laser capture microdissection of six distinct groups of tubular cross-sections followed by PCR and Illumina sequencing confirmed the presence of the *FGFR2* c.755C > G (p.Ser252Trp – Apert syndrome) (Fig. 3C,E) and *PTPN11* c.214G > C (p.Ala72Pro – Noonan syndrome) mutations in distinct enhanced MAGEA4-tubules (Fig. 3D,F), consistent with the geographic location of these specific variants identified by deep sequencing in the adjacent Tes2F slice (Fig. 3B). For the three other testes, FFPE blocks were not available.

## Discussion

We present a new broad-scale approach to studying clonal de novo germline mutations directly in human adult testes, the tissue where the majority of DNMs originate. By using massively parallel multiplex PCR and ultradeep sequencing followed by the implementation of a statistical prioritization calling strategy, we identified 61 different variants in a total of 111 mutation-positive testicular biopsies, 59 of which encode nonsynonymous substitutions (Table 1).

Several observations support the notion that the mutations identified are enriched for clonal events that are promoted by positive selection of mutant stem cells via the phenomenon of selfish spermatogonial selection. Out of the 61 validated variants (Table 1), 43 are located in five (*FGFR2*, *FGFR3*, *KRAS*, *PTPN11*, *RET*) of the six genes associated with strong prior experimental evidence for this process (Supplemental Table S1). As detailed in Table 1 and illustrated in Figure 2 and Supplemental Figure S3, the vast majority of variants identified across these five genes overlap with those observed in dominant congenital disorders and/or cancer, suggestive of a functional role via a gain-of-function mechanism. The most commonly observed individual mutation was *FGFR2* c.755C > G (p.Ser252Trp – Apert syndrome) detected in 23 biopsies. In this and other cases, the identification of identical variants in multiple neighboring testis biopsies (Fig. 1; Supplemental Fig. S2) is supportive of clonal expansion along the length of the seminiferous tubules, and in three cases, this process could be directly validated at a cellular level by visualizing the selfish expansion characterized by enhanced MAGEA4 staining in the adjacent testis block (Fig. 3; Maher et al. 2016a). The largest number of mutations was observed for *PTPN11* (encoding the SHP2 tyrosine phosphatase), in which we identified 20 different variants (across 33 biopsies) (Table 1; Fig. 2B). We observed 12 distinct variants located within the N-SH2 domain of SHP2, a region of the protein known to repress the catalytic phosphatase domain in its wild-type state (Neel et al. 2003), including each of the possible nucleotide substitutions at *PTPN11* c.215C encoding three distinct amino acids (p.Ala72Asp, p.Ala72Gly, and p.Ala72Val) that have been associated with Noonan syndrome or oncogenesis. The large number of different de novo variants is consistent with epidemiological data that concur that *PTPN11*-associated Noonan syndrome mutations have a high spontaneous birth prevalence (about one in 10,000 births) (Goriely and Wilkie 2012). We also identified two dinucleotide (tandem base) substitutions in *PTPN11*: Both the c.226_227delGAinsCT (p.Glu76Leu) (no. 30) and the c.1504_1505delTCinsAA (p.Ser502Lys) (no. 55) variants encode amino acid substitutions that, owing to the nature of the genetic code, cannot arise from single-nucleotide changes. These observations are reminiscent of other previously described selfish mutations encoded by double and triple substitutions, which in some cases were shown to result via a “double-hit” mechanism (Goriely et al. 2005; Goriely and Wilkie 2012; Giannoulatou et al. 2013). In humans, the de novo tandem mutation rate is estimated to be ∼0.3% of the single-nucleotide variant rate (Besenbacher et al. 2016); in this small set of 61 variants, we find an approximately 10-fold enrichment over the background rate.

Given this strong support for positive clonal selection of pathogenic variants in previously known selfish genes, the next question is whether the other 18 validated variants present in novel candidate genes might also signal the presence of selfish selection. We first excluded from consideration one variant, *NF1* c.2280G > A p.(Met760Ile) (no. 49), which presented with a different pattern of occurrence characterized by an extended geographical distribution across about one-third of the testis from individual Tes4, raising the possibility of an early post-zygotic (as opposed to adult-onset) mutational event (Supplemental Fig. S5). Although this *NF1* variant exhibits a high CADD score (24.6), has been reported in one case of lung cancer (Redig et al. 2016), and is located within the cysteine-serine–rich domain, a region where several missense mutations associated with breast cancer and neurofibromatosis have been identified (Koczkowska et al. 2018), its pathogenic status—and potential for positive selection—remains uncertain.

Of the remaining 17 variants, all but three are accounted for by six genes (*BRAF*, *CBL*, *MAP2K1*, *MAP2K2*, *RAF1*, and *SOS1*) encoding members of the RAS-MAPK pathway, among which nine variants have previously been reported in either congenital disorders or cancer (Table 1; Supplemental Fig. S3). Moreover, for several variants (BRAF p.Gly469Ala, MAP2K1 p.Lys57Asn and p.Gln56Pro, MAP2K2 p.Cys125Ser, RAF1 p.Ser257Leu and p.Pro261Ala), direct biochemical evidence of a dominant gain-of-function activity is available (Wan et al. 2004; Kobayashi et al. 2010; Van Allen et al. 2014; Arcila et al. 2015). In fact, only three validated variants (nos. 1, 2, 47), for which evidence of involvement in selfish selection is weak or can be ruled out, were found in genes (*APC*, *AKT3*, *LRP5*) that function outside the RTK-RAS-MAPK pathway (see Supplemental Note). Hence, although only 41.9% of the callable sequence of our panel comprised RTK-RAS-MAPK candidate genes, 95% (57/60) of the validated variants represented known or very likely pathogenic changes within members of this signaling pathway (*P* value = 4.233 × 10^−13^, two-tailed Fisher's exact test; logistic regression coefficient = 1.69, *P* value = 6.363 × 10^−15^) (see Supplemental Tables S7, S8; Supplemental Methods), reinforcing the proposal that activation of the RAS-MAPK pathway is the predominant mechanism underlying selfish spermatogonial selection (Goriely et al. 2003, 2009; Goriely and Wilkie 2012; Maher et al. 2016a). Mutations in other core cellular pathways screened here either may not be associated with positive selection in human testes or may lead to milder clonal expansions that will require more sensitive screening approaches to uncover. In addition, there may be positively selected mutations in other genes that were not targeted in this screen due to the limited panel size. Although it can be difficult to formally distinguish signals of selection from normal turnover/neutral drift dynamics whereby the random loss of some clones is compensated by the expansion of others over time (Klein et al. 2010b; Simons 2016; Zink et al. 2017), the highly significant enrichment of functionally significant (biochemically activating) mutations affecting a single signaling pathway argues against a neutral process.

Among the variants we identified, we observed a high proportion of strongly oncogenic mutations, with 23 of the 35 nonsynonymous variants reported in COSMIC (v82) having never been described as constitutional mutations (Table 1). Strong gain-of-function mutations would be more likely to promote efficient expansion of spermatogonial stem cells and result in larger clones that are easier to detect (Goriely et al. 2009; Giannoulatou et al. 2013). However, in order to be transmitted, the mutations must be compatible with formation of functional sperm and with embryonic development. We previously showed that tubules with spermatogonia harboring strongly oncogenic variants are associated with reduced numbers of post-meiotic cells (Maher et al. 2016a). This would represent a mechanism by which the testis “filters” the transmission of pathogenic mutations across generations, although proof of this concept would require the development of ultrasensitive assays to screen large numbers of sperm samples. It is noteworthy that despite the relative abundance of strongly oncogenic mutations in the adult male germline, testicular tumors originating from adult spermatogonia (spermatocytic tumors) are extremely rare, with an incidence of about one per million men and are mostly benign in nature (Ghazarian et al. 2015; Giannoulatou et al. 2017).

In this study, the majority of biopsies analyzed were from older donors. Given that both mutation occurrence and clonal sizes are anticipated to be age-related processes, we reasoned that older individuals’ testes would be more suitable for a discovery screen; i.e., they are more likely to show a higher frequency of random mutational events, among which selfish variants would have had time to expand to a clonal size amenable to direct detection. Hence, the age range of the testes analyzed in this study was highly skewed, with >90% of biopsies being sampled from four older individuals (aged 71–90 yr) and the remaining, Tes5J, being sourced from a 34-yr-old man. While for three of the older individuals we identified multiple mutation-positive biopsies (Fig. 1; Supplemental Fig. S2), Tes5J from the younger man contained only two mutation-positive biopsies—likely representing a single clonal event—carrying the oncogenic *CBL* c.1211G > A (p.Cys404Tyr) variant (at VAF 0.5%–0.6%), in keeping with the expectation that the prevalence and size of mutant clones increases with time (Fig. 1D). It was, however, surprising that no variants were detected in Tes3D, given the advanced age of the donor (87 yr). Although it is possible that this individual may have had a low propensity to accumulation of selfish mutations, a more likely explanation is that only a few atrophic seminiferous tubules with hypospermatogenesis were present in this testis, a phenomenon known as progressive tubular involution commonly described in elderly men (Paniagua et al. 1987). Unfortunately, as no tissue had been preserved for histological analysis (Supplemental Table S5), we were unable to determine the status of spermatogenesis in this testis.

Our study has several technical limitations. The majority of variants identified were present at VAFs <1%, close to the typical detection limits attributable to the error rates associated with DNA damage (10^−2^–10^−4^) (Arbeithuber et al. 2016; Chen et al. 2017), PCR (10^−4^–10^−6^) (Hestand et al. 2016; Potapov and Ong 2017), and Illumina sequencing (∼10^−3^) (Minoche et al. 2011; Salk et al. 2018). To account for such technical confounders, we employed a conservative custom statistical approach to determine the background error rate at each position and to prioritize variants (Supplemental Fig. S1). Although we confirmed variants with a frequency as low as 0.06% using this approach, the majority (81.8%) of the prioritized variants called in single amplicon at VAFs of 0.1%–0.2% (Tier 3) were false positives. In the 12 samples amplified and sequenced in duplicate, only seven of 15 variants were called in both replicates (Supplemental Table S4). The best predictor of true positives was the presence of a call in more than one amplicon (100% validation rate); for calls in single amplicons, the best predictor was the pathogenicity of the variant (17 of 18 [94.4%] pathogenic variants vs. five of 30 [16.7%] without prior disease association validated). Broad-scale approaches that target both DNA strands and use unique molecular indexes such as duplex sequencing (Kennedy et al. 2014) or smMIPs (used here to validate a subset of variants) (Hiatt et al. 2013) represent valuable alternatives to direct PCR amplification in future studies to reduce background errors (Salk et al. 2018). Overall, 14% of the designed amplicons did not pass QC (due to insufficient coverage and/or mapping error), which included those targeting candidate PAE mutations such as eight mutational hotspots in *FGFR3,* six in *PTPN11*, one in *RET*, and other key hotspots in *SKI* (Shprintzen-Goldberg syndrome), *SETBP1* (Schinzel-Giedion syndrome), and *AKT1* (Proteus syndrome, oncogenesis). Although considered to be the most frequently mutated nucleotide in the germline with a birth prevalence of about 1:30,000 (Bellus et al. 1995), we did not detect the *FGFR3* c.1138G > A or c.1138G > C achondroplasia-associated mutations due to exclusion of this region because of insufficient coverage (less than 5000×) (Supplemental Table S2; Supplemental Fig. S3E).

In summary, this work represents a new approach to studying DNMs directly in their tissue of origin. By utilizing the clonal nature of mutations that leads to focal enrichment, we circumvented the technical difficulties associated with calling DNMs in single sperm or the poor DNA quality associated with immunopositive tubules from FFPE material. In a single biopsy, a whole population of de novo mutations can be assessed. Studying mutations within the testis facilitates identification of mutations and pathways under positive selection in spermatogonia but that may be incompatible with life, either by impairing gamete differentiation and sperm production or by causing early embryonic lethality. Our approach reveals the prevalence and geographical extent of clonal mutations in human testes, suggesting that the aging male germline is a repository for functionally significant, often deleterious mutations. Based on an estimated total birth prevalence of DNMs causing developmental disorders of one in 295 (Deciphering Developmental Disorders Study 2017), such PAE mutations may contribute 5%–10% of the total burden of pathological mutations, depending on paternal age. Investigating the clonal nature of spontaneous testicular variants also provides insights into the regulation of the poorly studied human spermatogonial stem cell dynamics and into how spontaneous pathogenic mutations hijack homeostatic regulation in this tissue to increase their likelihood of transmission to the next generation.

## Methods

A schematic of the experimental protocol is presented in Supplemental Figure S1, and detailed methodology is provided in Supplemental Methods.

### Sample preparation and sequencing

Testes with no known phenotypic indicators and sourced with appropriate ethics approval from five men aged 34, 71, 83, 87, and 90 yr were cut into slices ∼3–5 mm thick, which were further dissected into 21–36 biopsies (Supplemental Table S5). DNA of sufficient quantity and quality was prepared from a total of 276 biopsies (Tes1D [34 biopsies], Tes2F [30 biopsies], Tes3D [32 biopsies], Tes4B-4G [153 biopsies from six slices], Tes5J [27 biopsies]). A 66.7-kb panel of 500 genomic regions in 71 genes was designed. The panel comprised mutational hotspots in the six established PAE genes; genes encoding other RTKs and members of the RAS-MAPK signaling pathway; genes in other pathways associated with spontaneous disorders that display narrow mutational spectra suggestive of gain-of-function effects but lacking epidemiological data for paternal age-effect; oncogenes commonly mutated in cancer, some of which are also associated with germline disorders; and regions of 10 “neutral-test” genes. Of note, a total of nine variants in the “neutral-test” set were long-listed following filtering (Supplemental Table S3), seven of which were in Tier 4. The two Tier 2 variants in the “neutral-test” set were rescreened and shown to be false-positive calls (Supplemental Note). Details of all targeted regions and primers used for amplification are provided in Supplemental Table S6.

The 500 target regions were amplified by massively parallel simplex PCR using the RainDance Thunderstorm target enrichment system. Droplets containing up to five primer pairs were merged with gDNA droplets to generate an average of 2 × 10^6^ droplets per sample (525,000 haploid genomes; average of one haploid genome per three to four droplets; about 1000 genomes/individual primer pair). Following the merge, libraries were PCR-amplified and purified, and tailed libraries for Illumina sequencing were constructed by PCR using a set of 8-bp barcoded adaptors (BC1-18). A total of 288 samples from 276 biopsies (12 biopsies were amplified in duplicate) (Supplemental Table S5) were amplified across six ThunderStorm enrichment chips (48 samples each) and subsequently ultradeep sequenced (about 22,000×) on two flow cells (16 lanes; 18 samples per lane) of Illumina HiSeq 2000 (2 × 100 bp).

### Sequence alignment, and variant calling and prioritization

Reads passing QC (on average 86% of reads) were aligned to the human genome (hg19) using BWA-MEM version 0.7.10 (Li 2013) with default parameter settings. As only genomic regions of well-characterized disease genes were interrogated in this study, realignment of the data to the GRCh38 assembly would not significantly affect the identification of low-level de novo variants. Pileup was then performed for each amplicon independently. After trimming of primer sequences, reads with more than 10 nonreference bases were removed (<1% of coverage on average). To avoid double-counting reads at positions where read 1 and read 2 overlapped, only the base with the higher quality was considered. To reduce false-positive calls, only variants supported by at least 10 reads were called. To account for the technical confounders, the data were normalized (accounting for flow cell, lane, and average base quality at each position) using a simple linear model, and the median effect was removed from each lane to reduce the background signal.

Each nonreference nucleotide at each genomic position across the 288 samples was tested independently in each amplicon that passed QC (supplemental custom pipeline; https://github.com/zd1/raindance). Variant prioritization was performed using a *P*-value cutoff of –log_10_*P* > 20, which resulted in a total of 19,625 genomic positions with at least one nonreference call. Further filtering was performed to remove potential sources of artifacts: samples or amplicons with an excessive number of variants (Supplemental Fig. S6), calls positioned 1 base from the amplification primer's 3′-end, calls with a maximum VAF of ≥3%, and positions with a median depth coverage across all samples below 5000× (Supplemental Table S2). This resulted in a total of 5729 calls (5659 distinct variants) at 5421 positions (in a total of 431 amplicons, corresponding to 51.5 kb of unique genomic sequence, across 67 genes), the majority (90.2%) of which were made in a single amplicon and sample. As singleton calls were more likely to represent PCR or sequencing artifacts, we further prioritized calls made in two or more samples and/or present in overlapping amplicons. Variant calls showing evidence of library-specific batch or sequence misalignment effects were excluded from further analysis. The remaining 115 variants at 105 genomic positions were annotated with ANNOVAR version 2015Jun17 (Wang et al. 2010) (for full details of the 115 variants, see Supplemental Table S3). If a variant was covered by more than one amplicon or was present in a replicated biopsy, the VAFs presented in Table 1 and all figures represent the mean allele frequency of the called variants.

### Variant validation

DNA from at least one putative-positive biopsy sample and at least eight control samples (unrelated blood gDNA and gDNA from other testicular biopsies) was screened by PCR amplification using different primers from those used in the RainDance experiment or by smMIPs capture and ultradeep sequencing (about 30,000×) using Illumina MiSeq 300v2 (PCR) or 150v3 (smMIP) kits (primer and smMIP details in Supplemental Table S6). Immunohistochemical staining of FFPE testis slices to identify tubules with enhanced spermatogonial MAGEA4 staining, followed by laser capture microdissection and DNA extraction, was performed as described (Maher et al. 2016a). DNA was subsequently amplified by PCR using CS-tagged primers and barcoded for Illumina MiSeq 300v2 sequencing.

## Data access

Raw sequencing FASTQ files from this study have been submitted to the European Nucleotide Archive (ENA; https://www.ebi.ac.uk/ena) under accession no. PRJEB28332.

## Competing interest statement

Z.D. is an employee of Genomics. His involvement in the conduct of this research was solely in his former capacity as a Statistical Geneticist at the University of Oxford.

## Supplementary Material

Supplemental Material
