# Supplementary material for: Selfish mutations dysregulating RAS-MAPK signaling are pervasive in aged human testes
Source: Genome Res. 2018 Dec;28(12):1779–90. doi: 10.1101/gr.239186.118 (PMC6280762; doi:10.1101/gr.239186.118)
Supplement: Supplemental Material [file supp_28_12_1779__index.html]

Selfish mutations dysregulating RAS-MAPK signaling are pervasive in aged human testes — Selfish mutations dysregulating RAS-MAPK signaling are pervasive in aged human testes — Supplemental Material 

# Selfish mutations dysregulating RAS-MAPK signaling are pervasive in aged human testes

## Supplemental Material

- Supplemental\_File.pdf
- Supplemental\_Table\_S2.xlsx
- Supplemental\_Table\_S3.xlsx
- Supplemental\_Table\_S4.xlsx
- Supplemental\_Table\_S5.xlsx
- Supplemental\_Table\_S6.xlsx
- Supplemental\_Custom\_pipeline.zip
