# Supplementary material for: Selfish mutations dysregulating RAS-MAPK signaling are pervasive in aged human testes
Source: Genome Res. 2018 Dec;28(12):1779–90. doi: 10.1101/gr.239186.118 (PMC6280762; doi:10.1101/gr.239186.118)
Supplement: Supplemental Material [file supp_gr.239186.118_Supplemental_File.pdf]

SUPPLEMENTAL FILE

Selfish mutations dysregulating RAS-MAPK signaling are pervasive in aged human testes

Geoffrey J. Maher¶, Hannah K. Ralph¶, Zhihao Ding¶, Nils Koelling, Hana Mlcochova, Eleni Giannoulatou, Pawan Dhami, Dirk S. Paul, Stefan H. Stricker, Stephan Beck, Gilean McVean, Andrew OM Wilkie, Anne Goriely

Table of Contents

File	Page
Supplemental Fig S1 – Schematic of experimental design	2
Supplemental Fig S2 – Distribution of mutations in slices Tes4B-4G from individual 4	3
Supplemental Fig S3 – A-M Individual gene plots showing the location of spontaneous mutations identified in testicular biopsies for AKT3 (A), APC (B), BRAF (C), CBL (D), FGFR3 (E), KRAS (F), LRP5 (G), MAP2K1 (H), MAP2K2 (I), NF1 (J), RAF1 (K), RET (L), and SOS1 (M).	4-17
Supplemental Fig S4 - Variant allele frequencies of *KRAS* c.182A>G (p.Gln61Arg) and *LRP5* c.291C>T (p.Ala97Ala) in all 276 biopsy samples.	18
Supplemental Fig S5 – Heatmap of *NF1* c.2280G>A and *KRAS* c.35G>A.	19
Supplementary Fig S6 – Number of variants called in each sample, organized by substitution type.	20
Supplemental Table S1 – Literature review showing loci with evidence for selfish selection	21
Supplemental Table S2 – Coverage analysis of 500 amplicons	Separate .xlsx file
Supplemental Table S3 – Table of prioritized calls (Tiers 1, 2, 3, 4)	Separate .xlsx file
Supplemental Table S4 – Variant calls in replicate samples	Separate .xlsx file
Supplemental Table S5 – Sample information	Separate .xlsx file
Supplemental Table S6 – Primers and smMIPs	Separate .xlsx file
Supplemental Table S7 – Details of the different gene categories on the targeted panel (for the 431 amplicons that passed QC)	22
Supplemental Table S8 – Regression coefficients describing the relative impact of several factors/variables as predictors of validated variants (ordered by P values).	23
Supplemental Note	24
Supplemental Methods	25

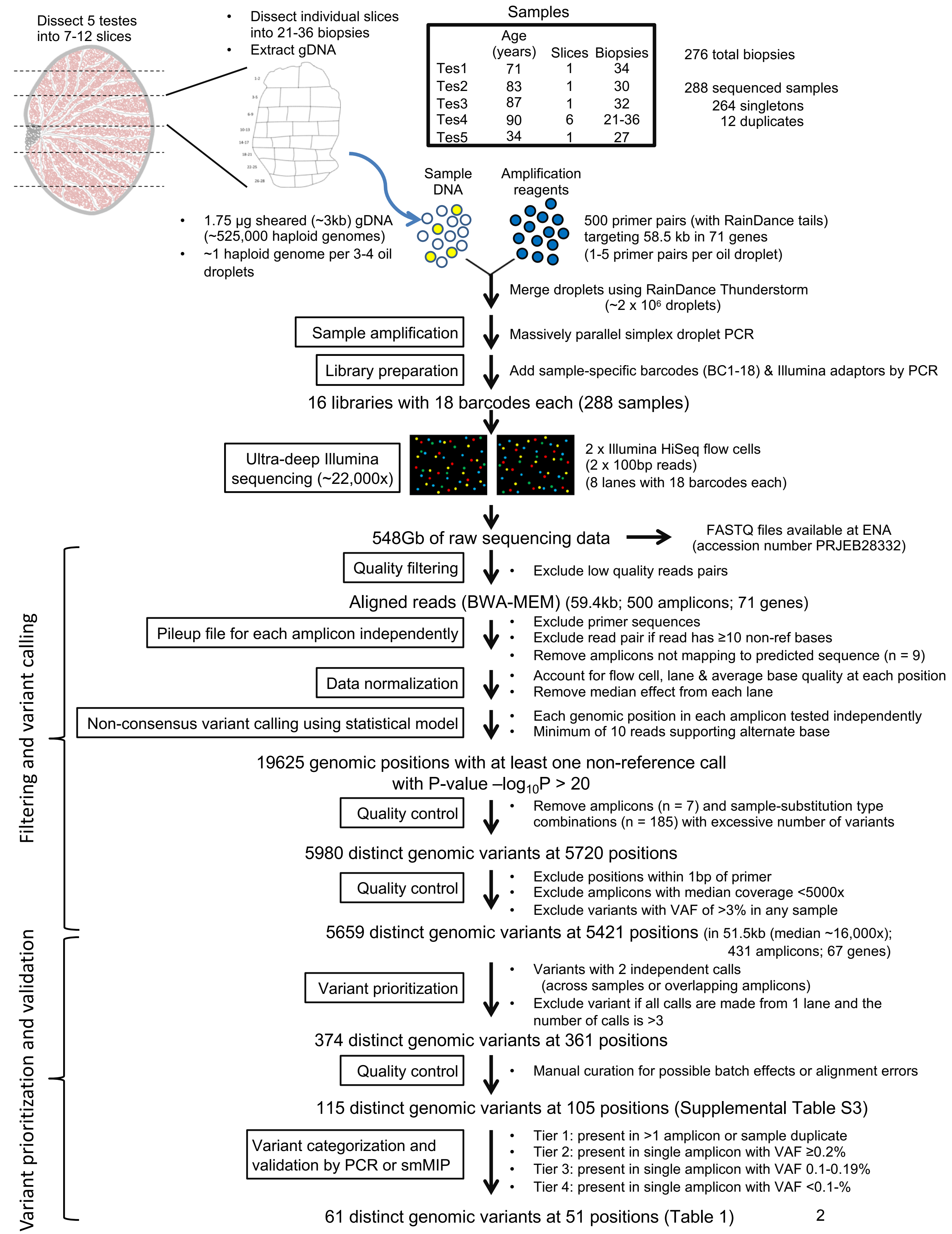

Supplemental Fig S1 – Schematic of experimental design

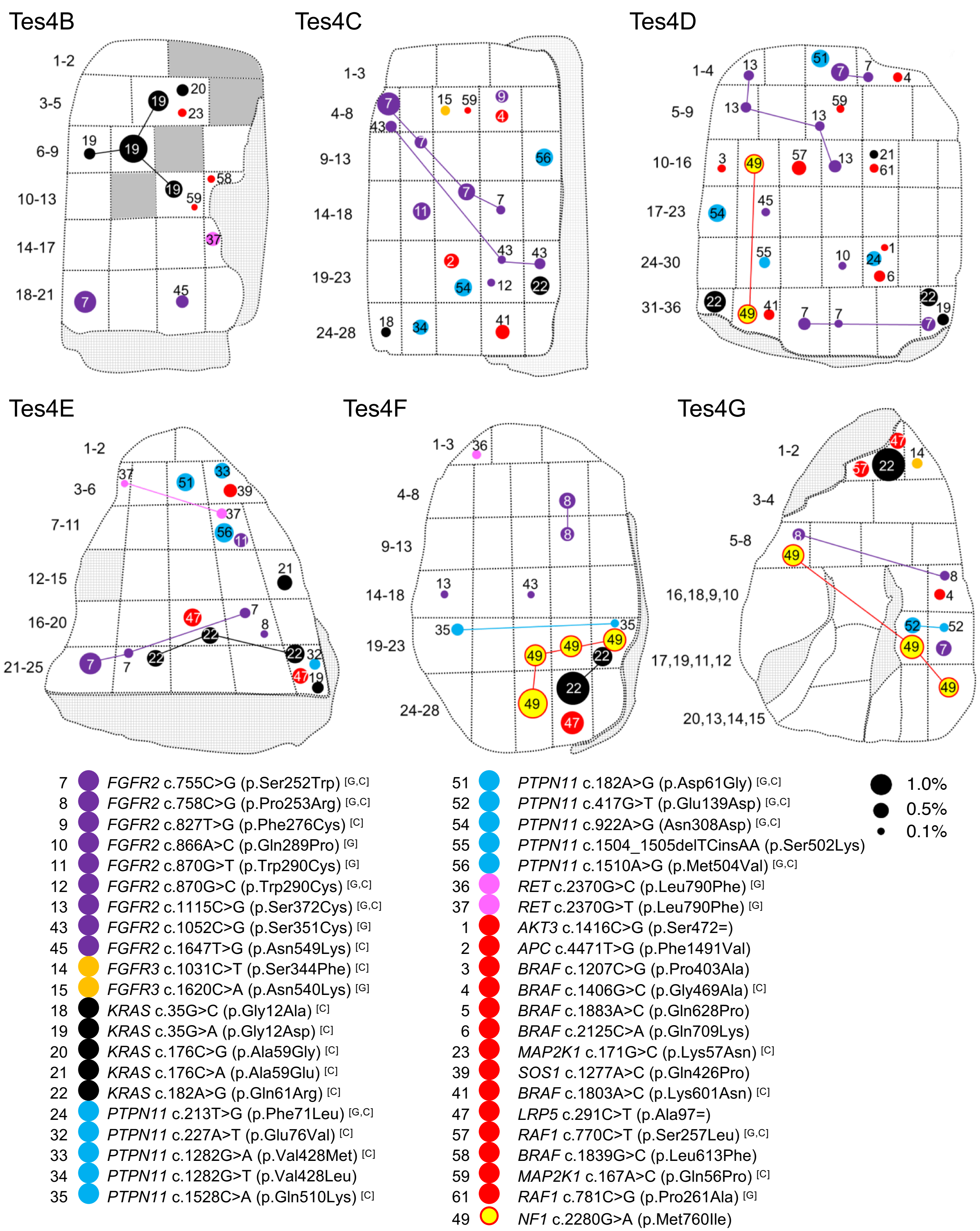

Supplemental Fig S2. Distribution of mutations in slices Tes4B-4G from individual 4. Testicular biopsy numbers are located outside and to the left of each testis slice. Each variant has a distinct number (as listed in Table 1) and is colored according to gene: *FGFR2* (purple), *FGFR3* (orange), *KRAS* (black), *PTPN11* (blue), *RET* (pink), newly associated gene (red), NF1 mosaic (yellow with red surround). The size of each circle is proportional to the mutation frequency. Lines connect biopsies in the same slice with identical mutations; in cases where more than two biopsies are positive, the path of the clone has been arbitrarily drawn. Solid grey regions represent biopsies that were not sequenced due to quality control issues. Gridded grey regions represent non-tubular regions of tissue. Variants are numbered in order of Tier: Tier 1 (1-39), Tier 2 (40-57), Tier 3 (58-61). Letters in brackets refer to variants associated with germline disorders [G] and/or reported in COSMIC database [C].

Supplemental Fig S3: Individual gene plots showing the location of spontaneous mutations identified in testicular biopsies for AKT3 (A), APC (B), BRAF (C), CBL (D), FGFR3 (E), KRAS (F), LRP5 (G), MAP2K1 (H), MAP2K2 (I), NF1 (J), RAF1 (K), RET (L), and SOS1 (M).
(Panel I) Validated variants (with VAF on y-axis) positioned along the amino acid sequence of the relevant protein (*x*-axis, see Panel V). (Panel II) Location and size of amplicons used to sequence main hotspots of the relevant genes are plotted on the *x*-axis. Median coverage per amplicon is plotted on the *y*-axis. Line indicates coverage cut-off of 5,000x. (Panel III) Number of reported constitutional variants encoding amino acid substitutions associated with developmental disorders (sqrt scale). (Panel IV) Number of reported somatic amino acid substitutions in cancer (COSMIC v82). (Panel V) Protein domains. Annotations are based on the transcripts' accession numbers listed in the Supplemental Methods.

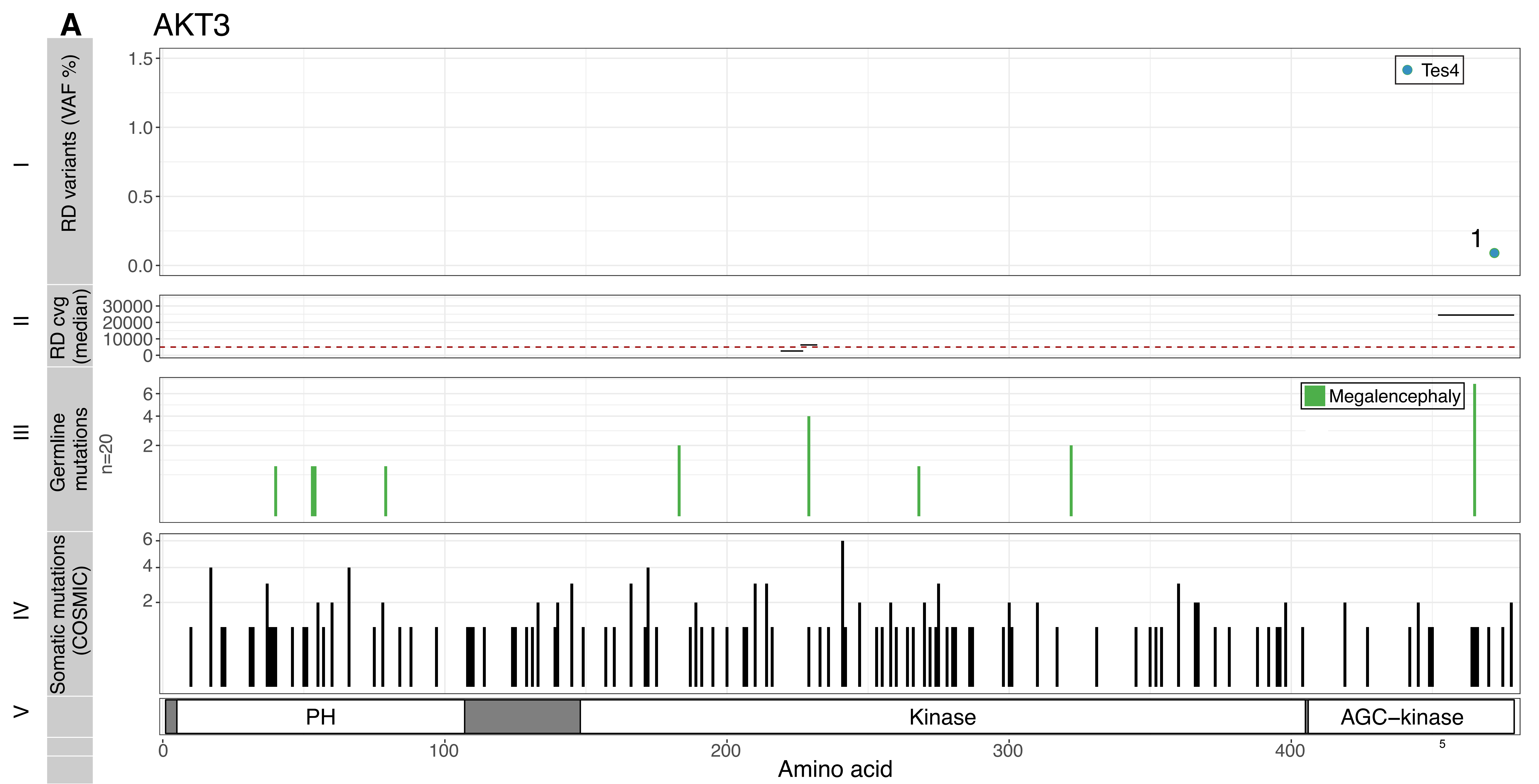
A
AKT3
I
II
III
IV
V
RD variants (VAF %)
Tes4
1
RD cvg (median)
Germline mutations
n=20
Megalencephaly
Somatic mutations (COSMIC)
PH
Kinase
AGC−kinase
Amino acid

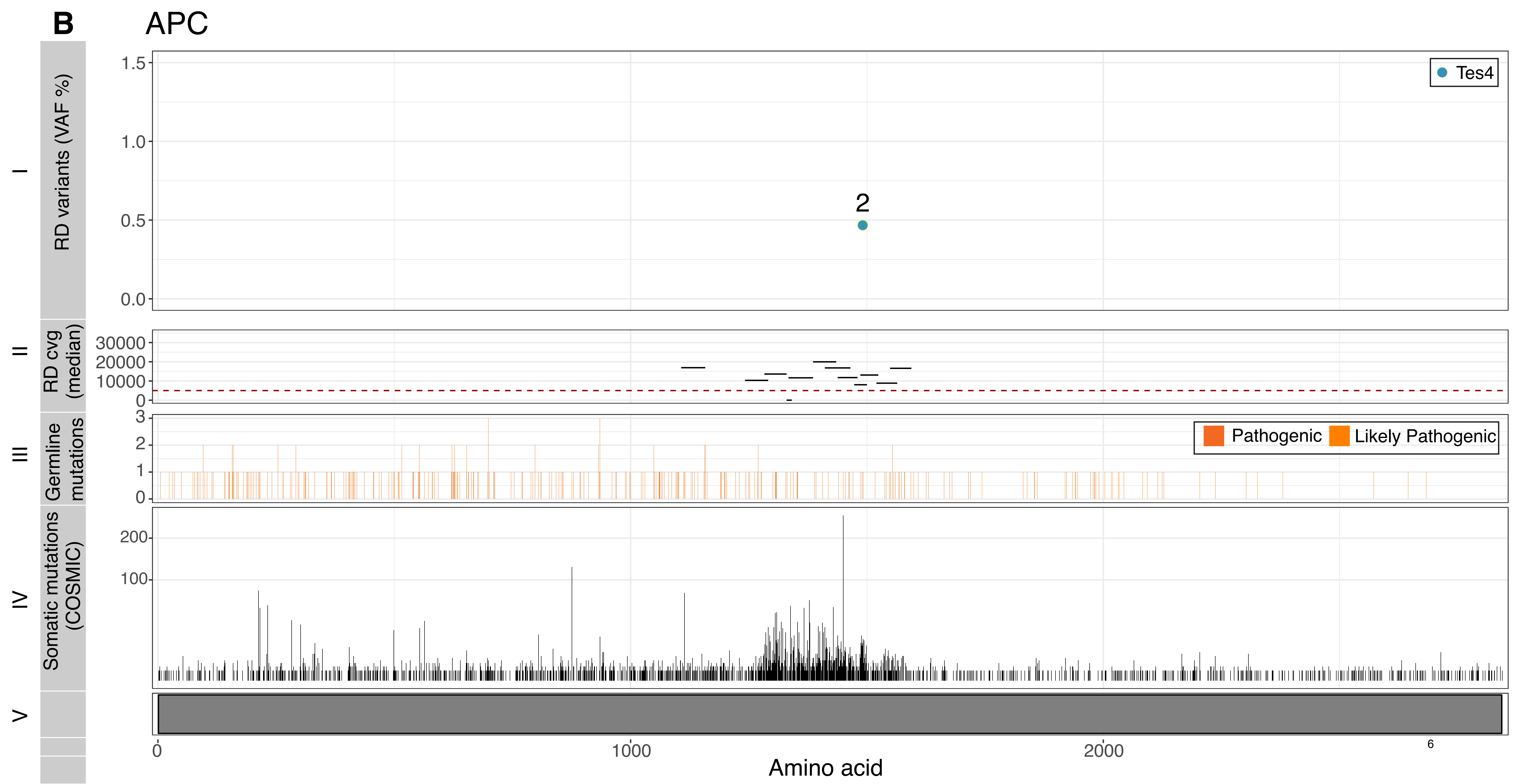
B
APC
I
RD variants (VAF %)
1.5
1.0
0.5
0.0
Tes4
2
II
RD cvg (median)
30000
20000
10000
0
III
Germline mutations
3
2
1
0
Pathogenic
Likely Pathogenic
IV
Somatic mutations (COSMIC)
200
100
V
0
1000
2000
Amino acid

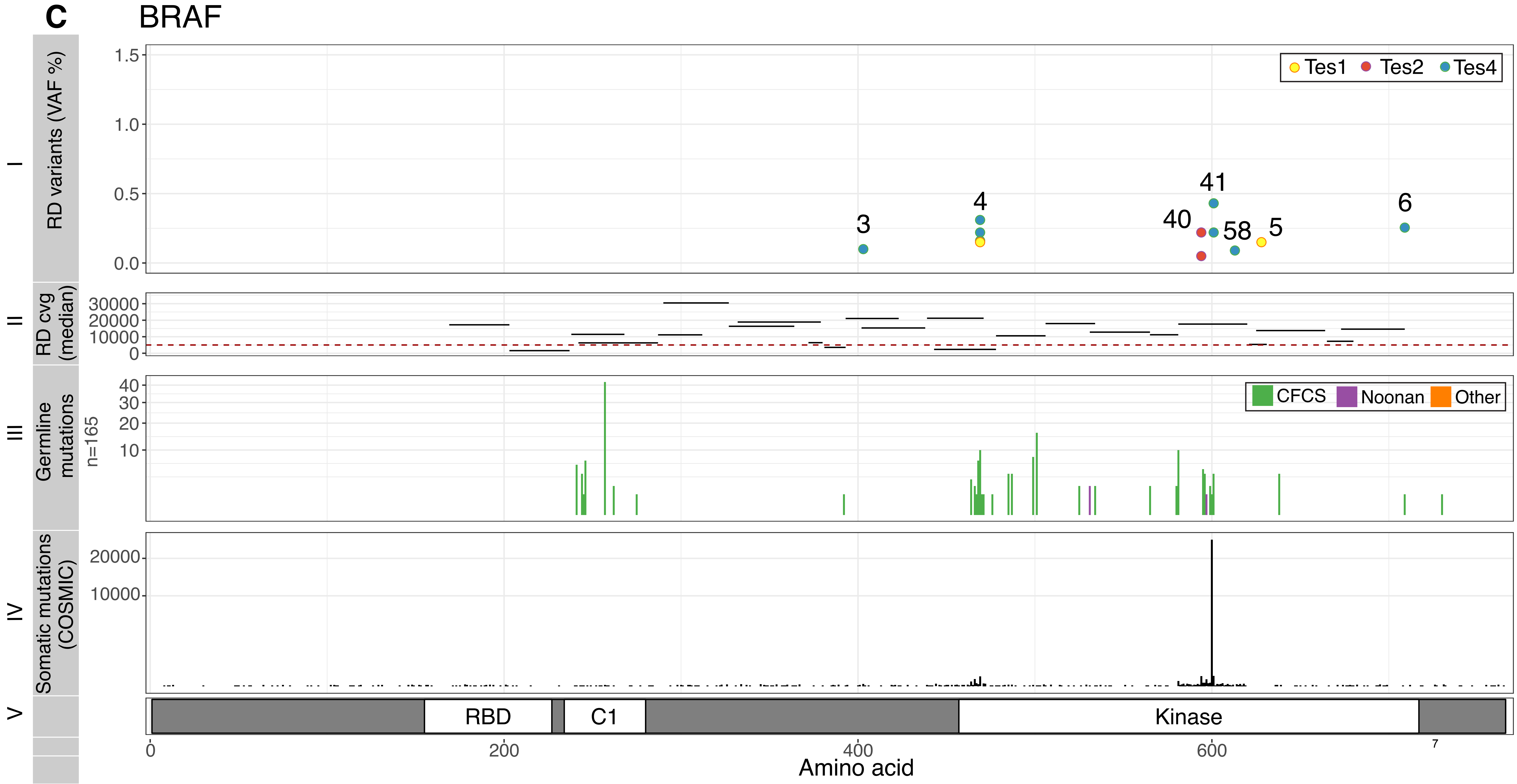
C
BRAF
I
RD variants (VAF %)
1.5
1.0
0.5
0.0
Tes1
Tes2
Tes4
3
4
41
40
58
5
6
II
RD cvg (median)
30000
20000
10000
0
III
Germline mutations
n=165
40
30
20
10
CFCS
Noonan
Other
IV
Somatic mutations (COSMIC)
20000
10000
V
RBD
C1
Kinase
0
200
400
600
Amino acid

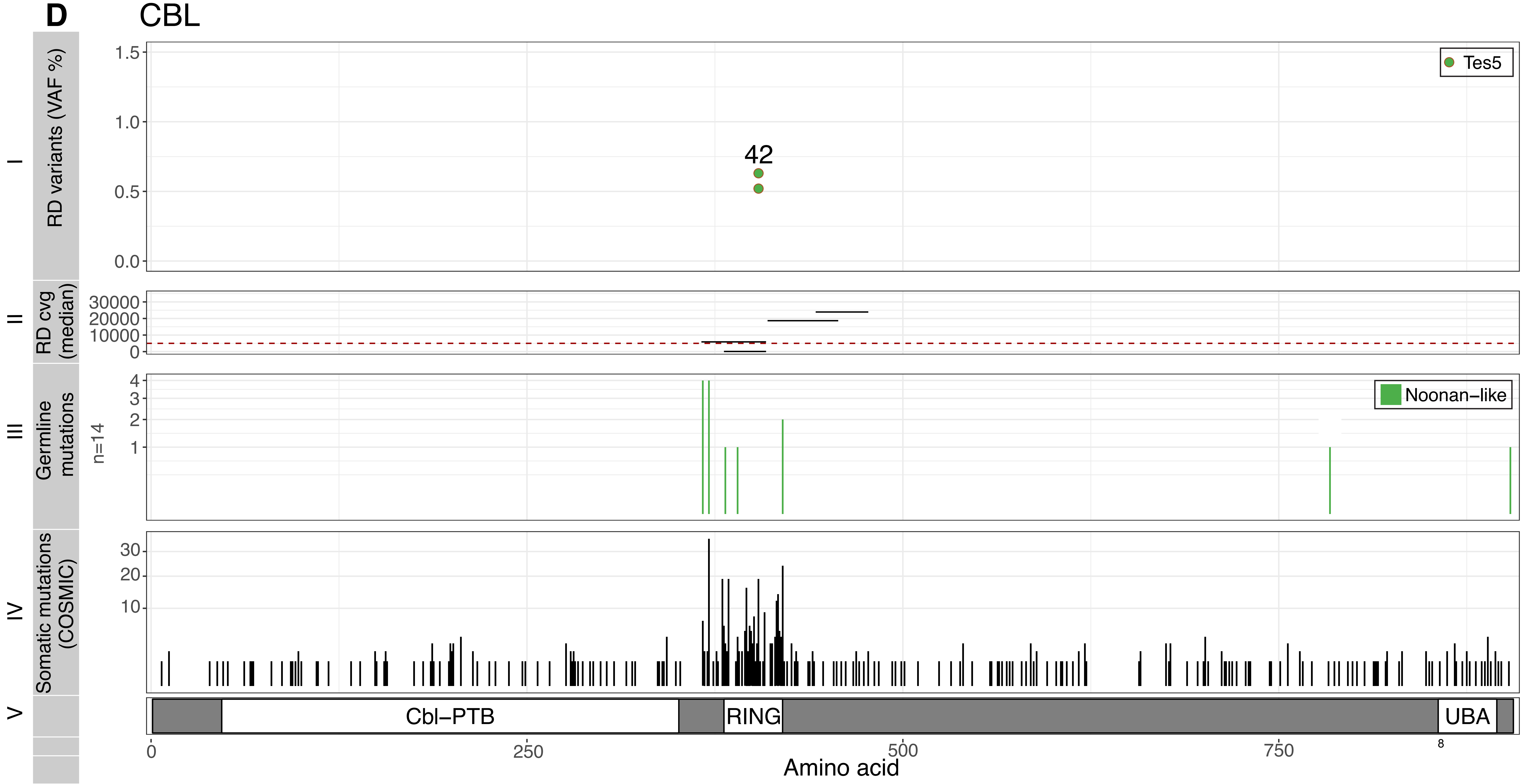
D
CBL
I
RD variants (VAF %)
Tes5
42
II
RD cvg (median)
III
Germline mutations
n=14
Noonan−like
IV
Somatic mutations (COSMIC)
V
Cbl−PTB
RING
UBA
Amino acid

E

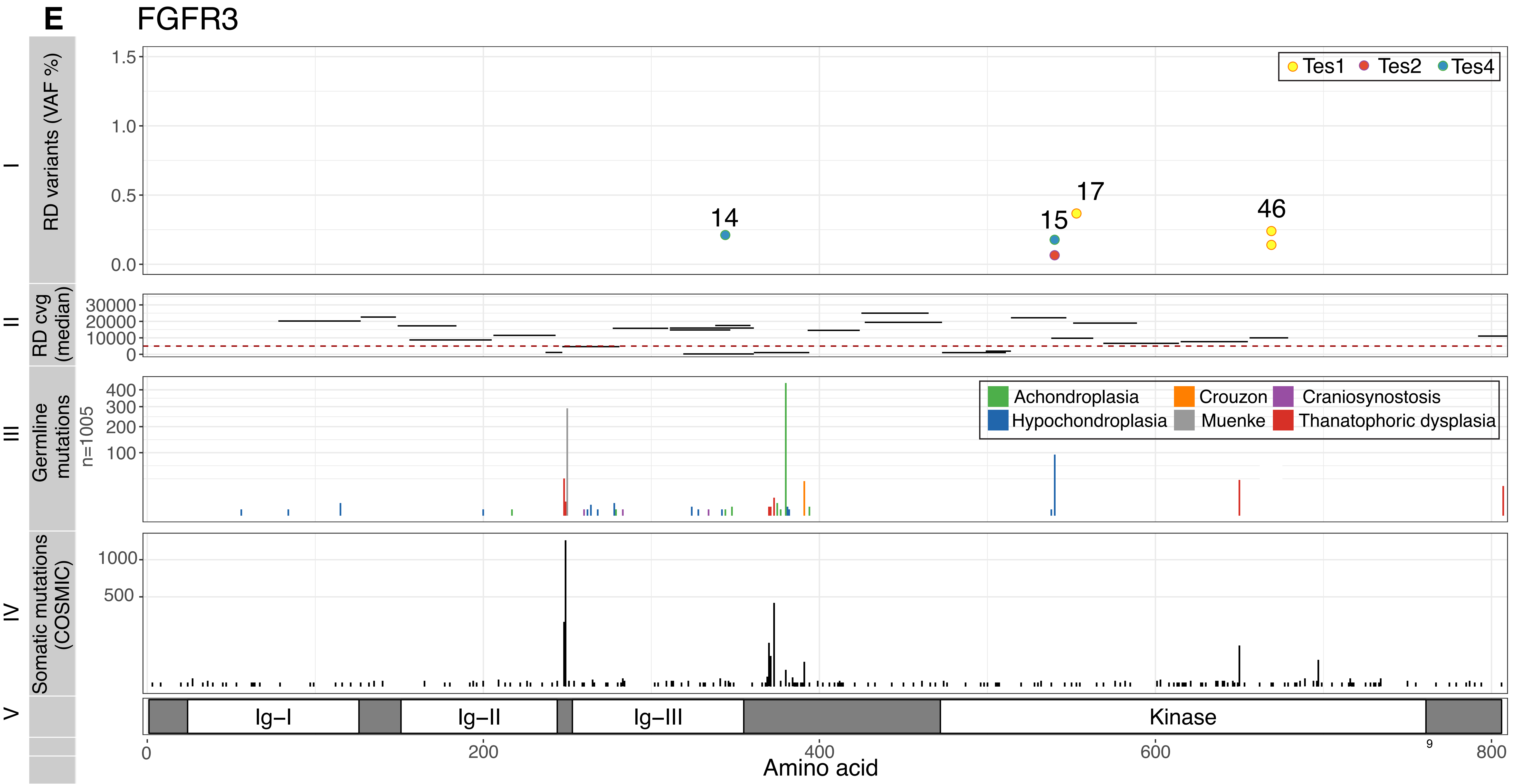

FGFR3
I
RD variants (VAF %)
1.5
1.0
0.5
0.0
Tes1
Tes2
Tes4
14
15
17
46
II
RD cvg (median)
30000
20000
10000
0
III
Germline mutations
n=1005
400
300
200
100
Achondroplasia
Crouzon
Craniosynostosis
Hypochondroplasia
Muenke
Thanatophoric dysplasia
IV
Somatic mutations (COSMIC)
1000
500
V
Ig–I
Ig–II
Ig–III
Kinase
0
200
400
600
800
Amino acid

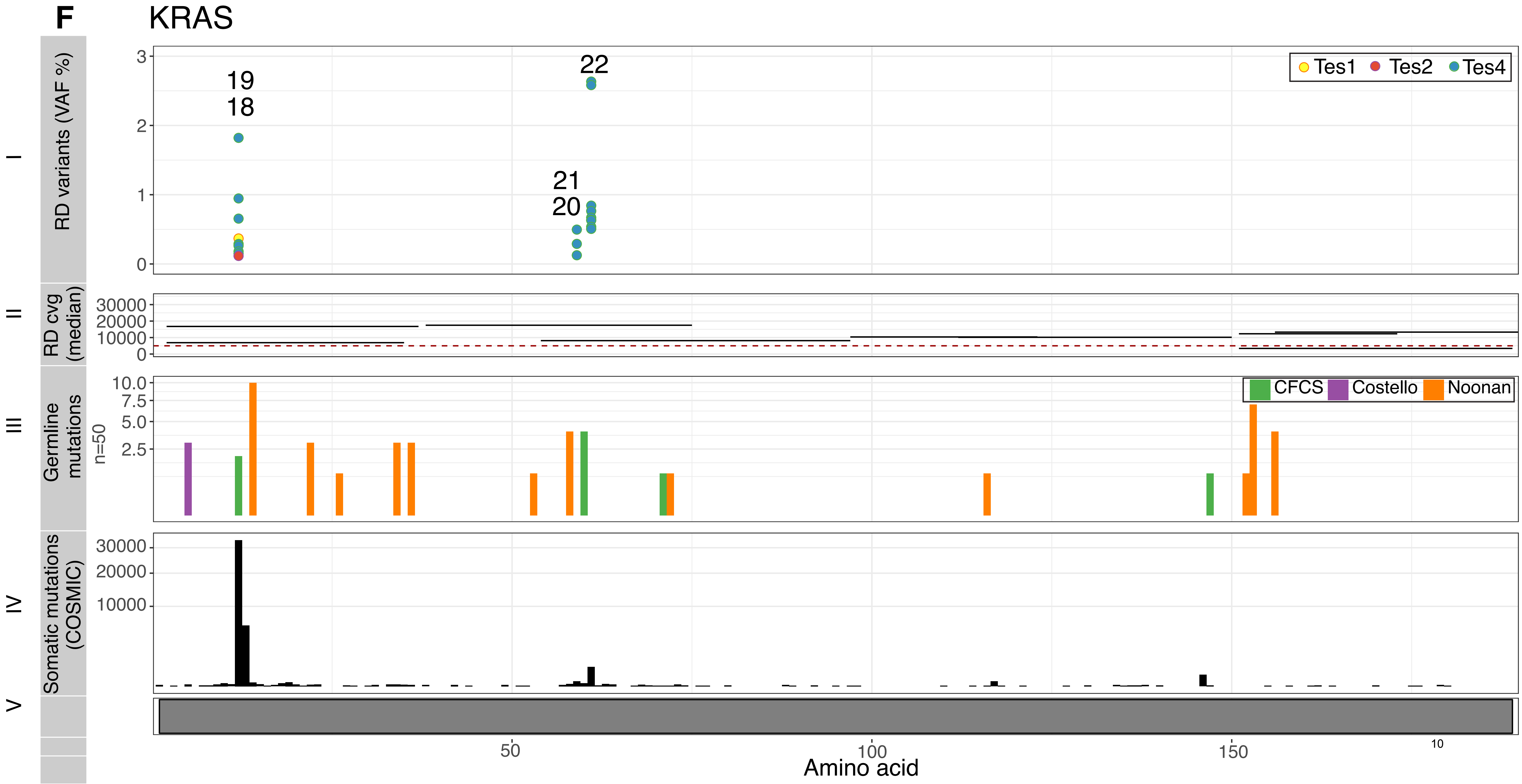
F
KRAS
I
II
III
IV
V
RD variants (VAF %)
3
2
1
0
19
18
22
21
20
Tes1
Tes2
Tes4
RD cvg (median)
30000
20000
10000
0
Germline mutations
n=50
10.0
7.5
5.0
2.5
CFCS
Costello
Noonan
Somatic mutations (COSMIC)
30000
20000
10000
50
100
150
Amino acid

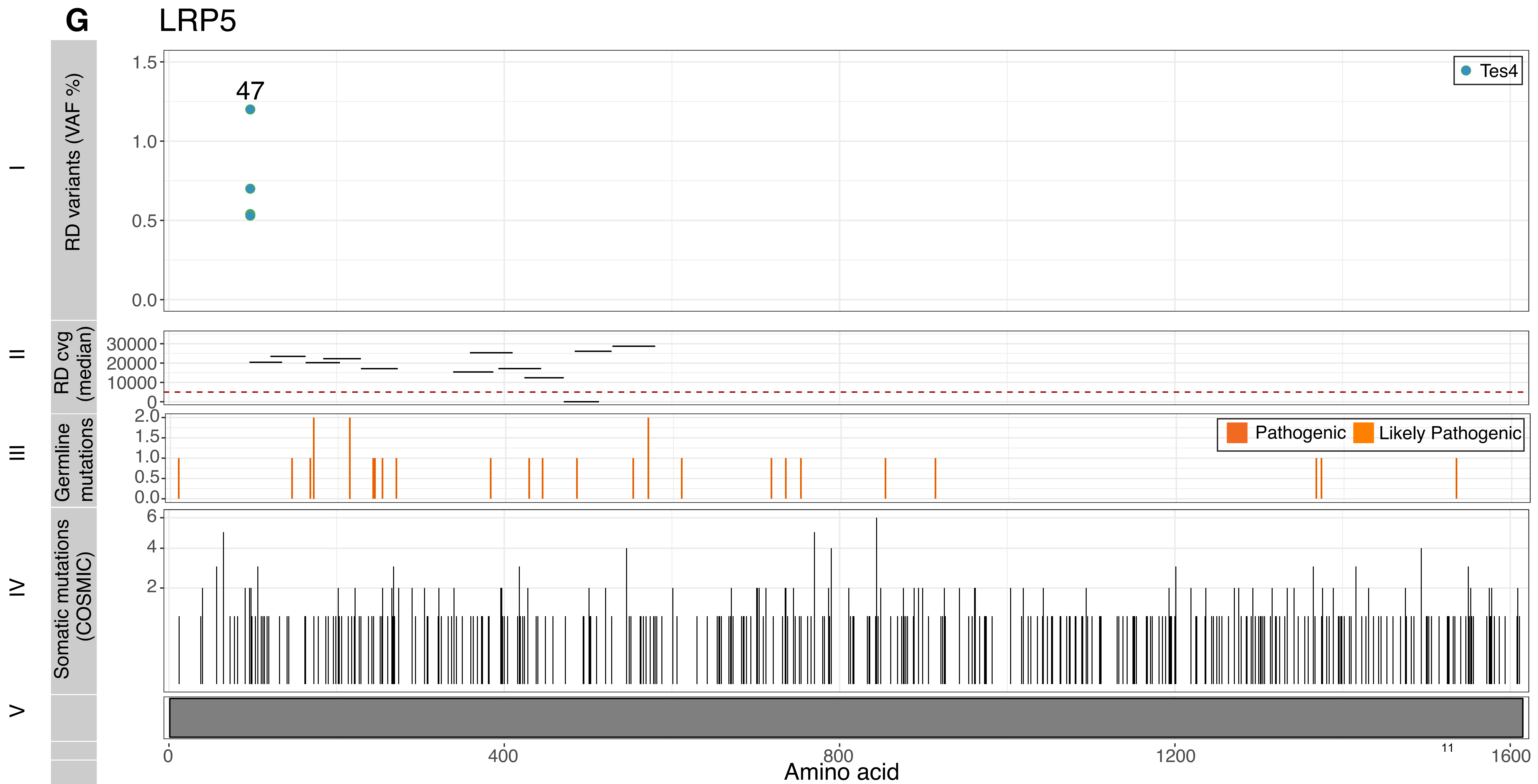

G
LRP5
I
II
III
IV
V
RD variants (VAF %)
RD cvg (median)
Germline mutations
Somatic mutations (COSMIC)
47
Tes4
Pathogenic
Likely Pathogenic
Amino acid

H

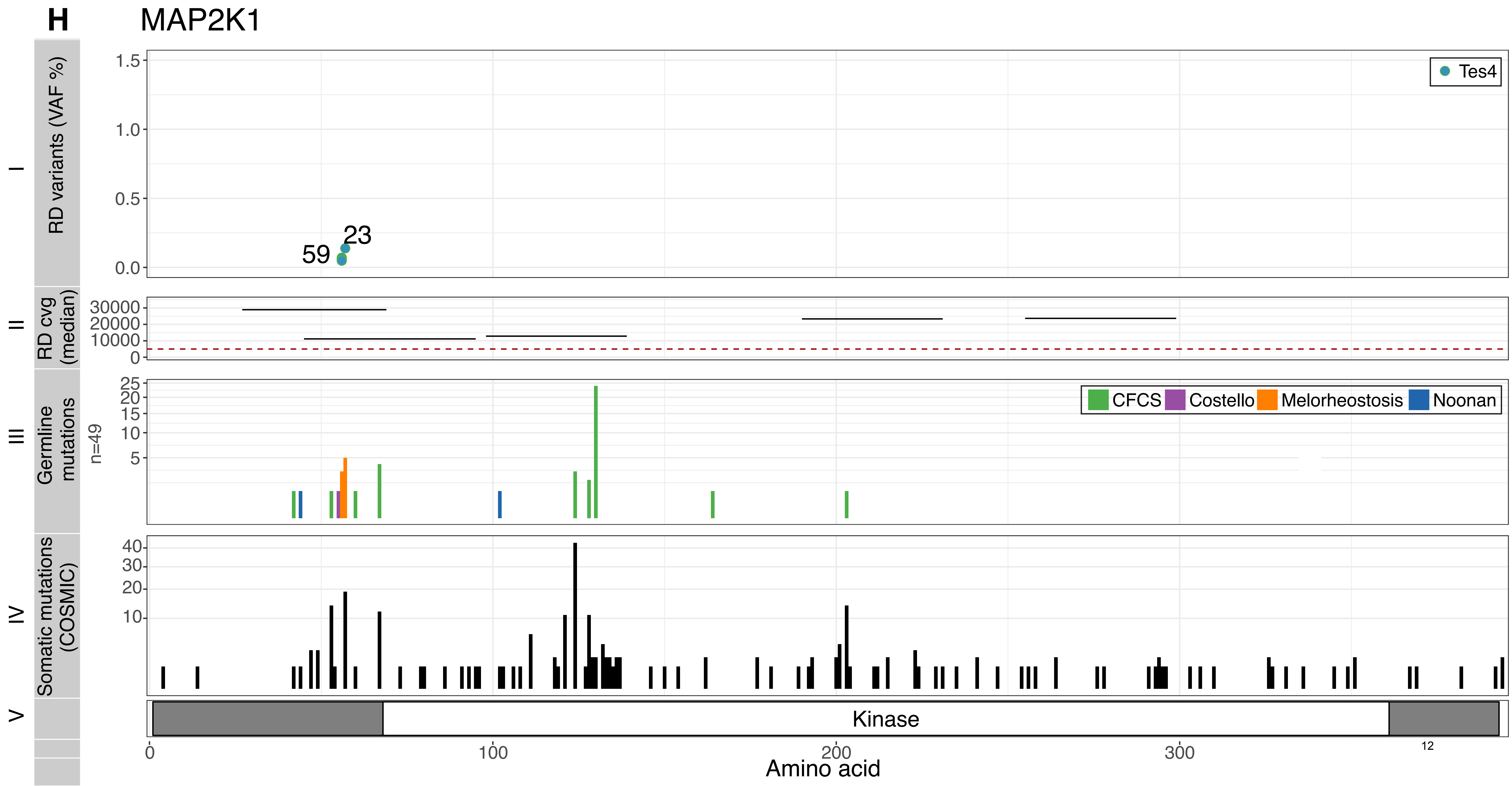
MAP2K1
I
II
III
IV
V
RD variants (VAF %)
1.5
1.0
0.5
0.0
Tes4
23
59
RD cvg (median)
30000
20000
10000
0
Germline mutations
n=49
25
20
15
10
5
CFCS
Costello
Melorheostosis
Noonan
Somatic mutations (COSMIC)
40
30
20
10
Kinase
0
100
200
300
Amino acid

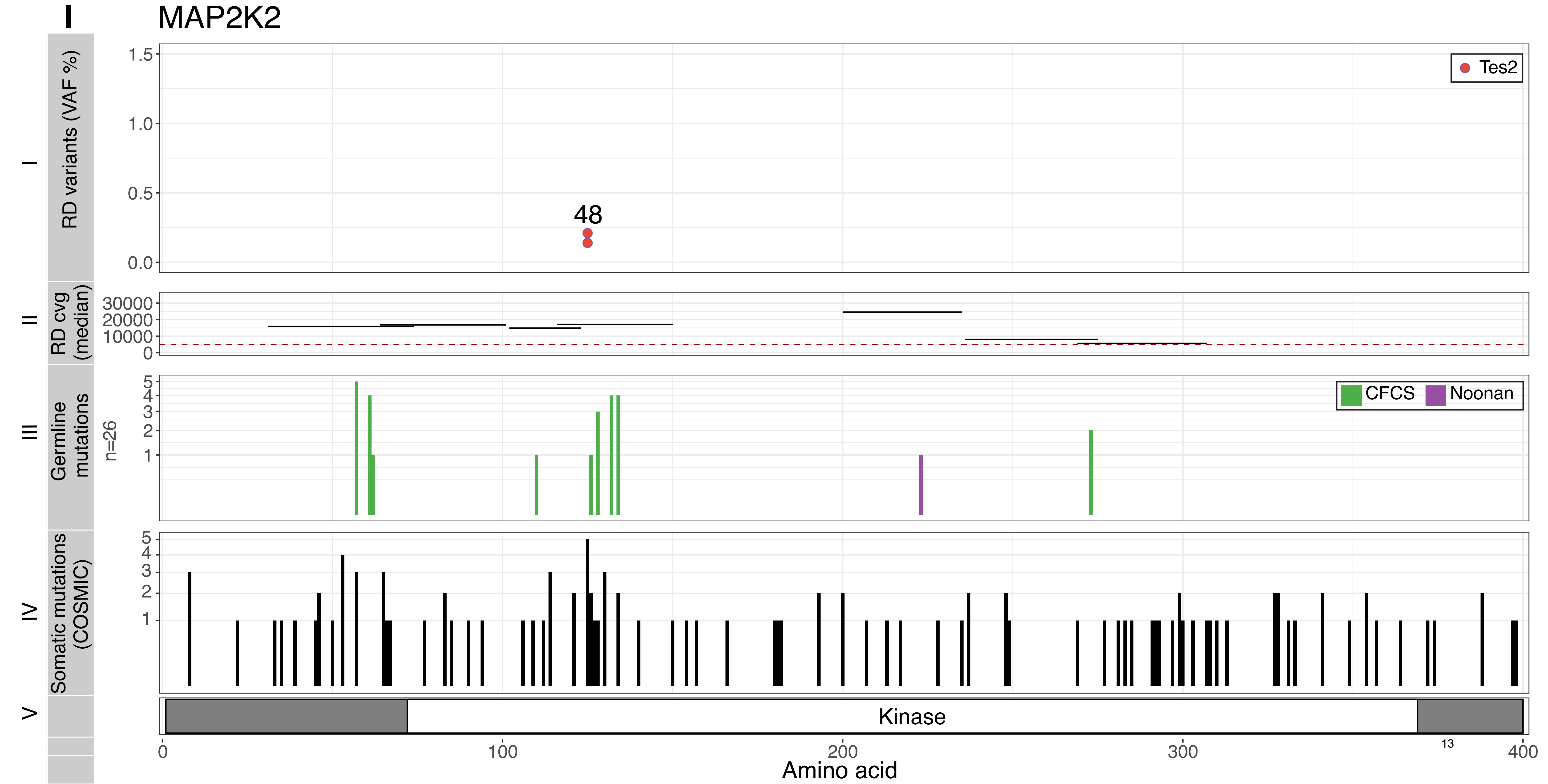
I
MAP2K2
I
RD variants (VAF %)
1.5
1.0
0.5
0.0
Tes2
48
II
RD cvg (median)
30000
20000
10000
0
III
Germline mutations
n=26
CFCS
Noonan
IV
Somatic mutations (COSMIC)
V
Kinase
0
100
200
300
400
Amino acid

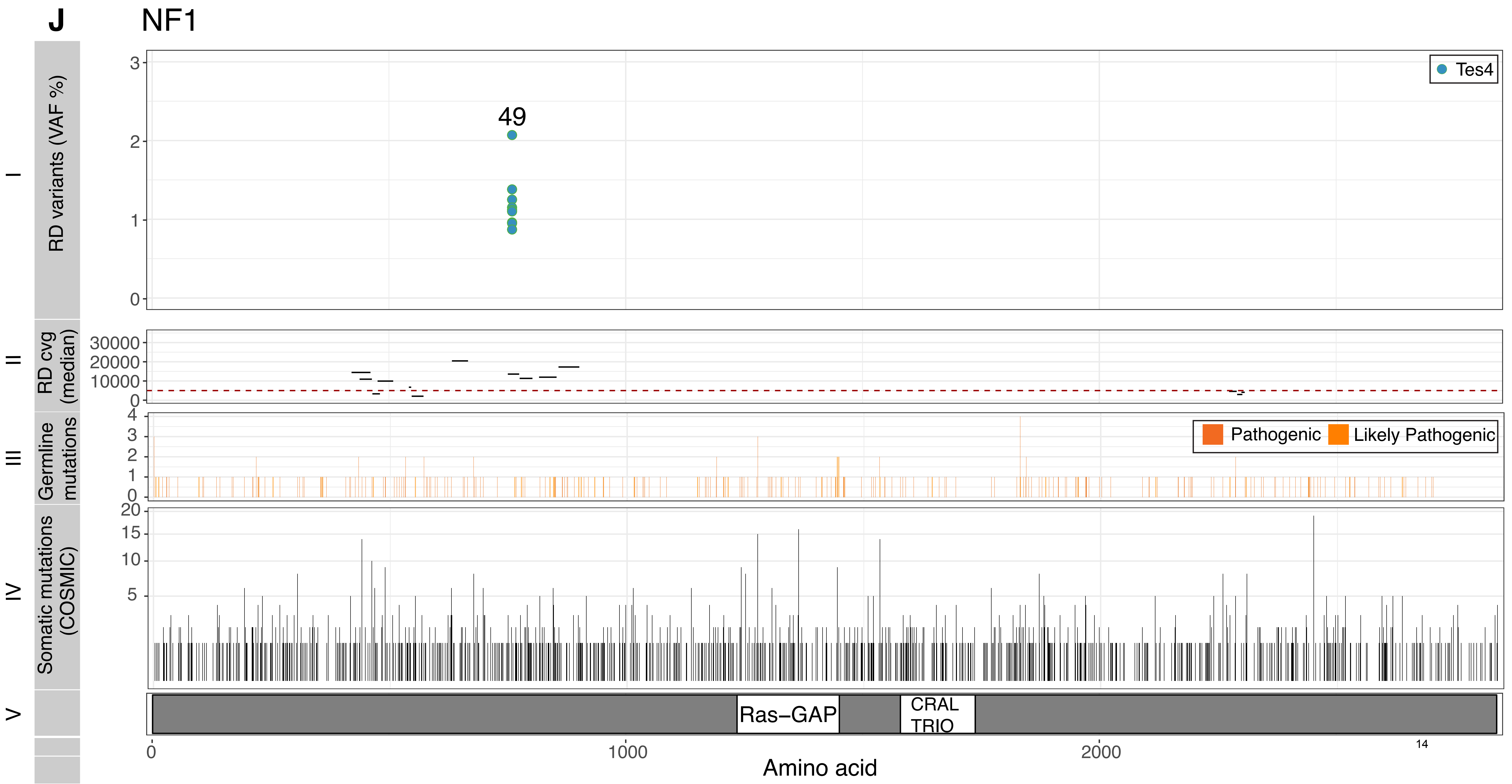

J
NF1
I
RD variants (VAF %)
3
2
1
0
49
Tes4
II
RD cvg (median)
30000
20000
10000
0
III
Germline mutations
4
3
2
1
0
Pathogenic
Likely Pathogenic
IV
Somatic mutations (COSMIC)
20
15
10
5
V
Ras−GAP
CRAL TRIO
0
1000
2000
Amino acid

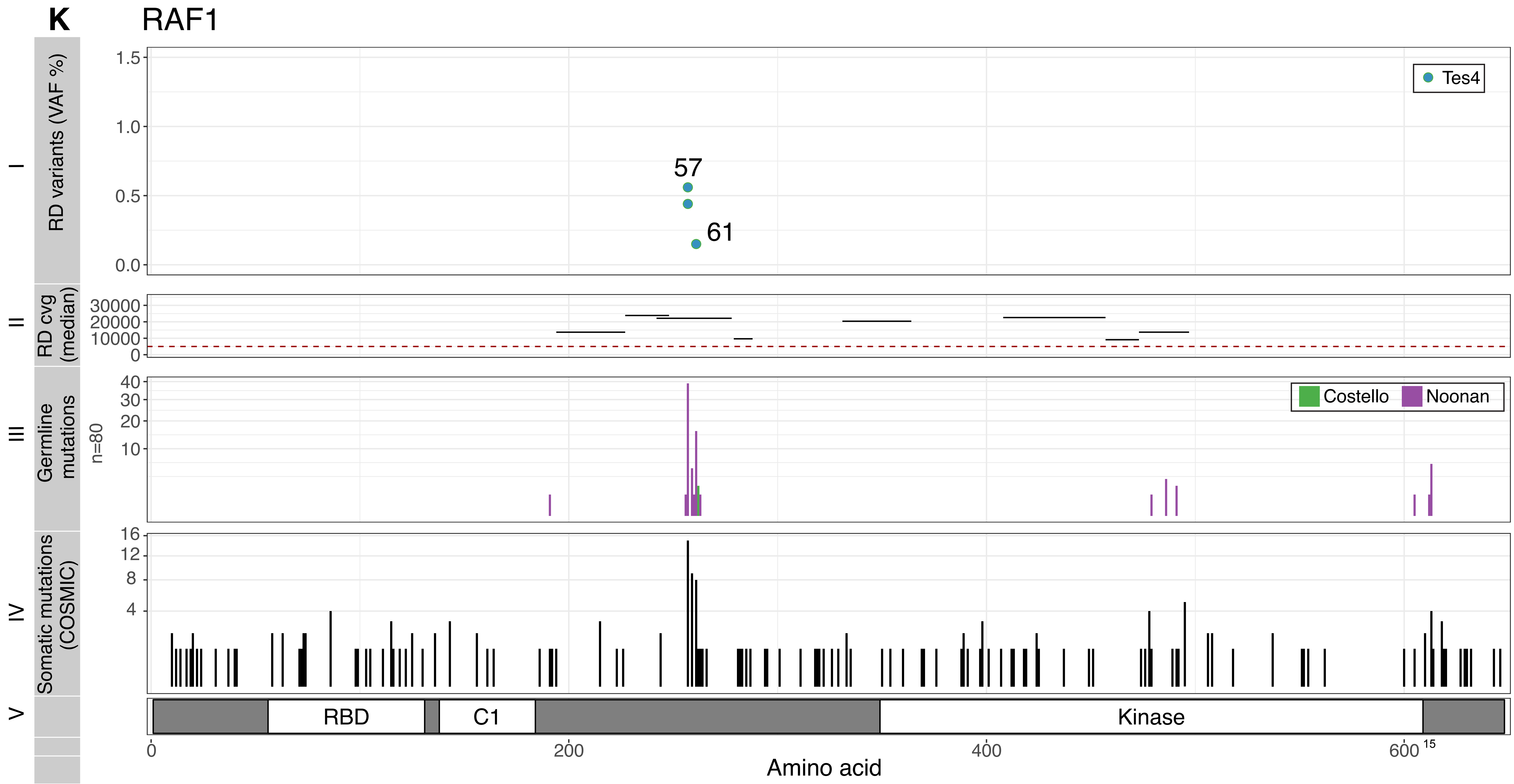
K
RAF1
I
RD variants (VAF %)
1.5
1.0
0.5
0.0
57
61
Tes4
II
RD cvg (median)
30000
20000
10000
0
III
Germline mutations
n=80
40
30
20
10
Costello
Noonan
IV
Somatic mutations (COSMIC)
16
12
8
4
V
RBD
C1
Kinase
0
200
400
600
Amino acid

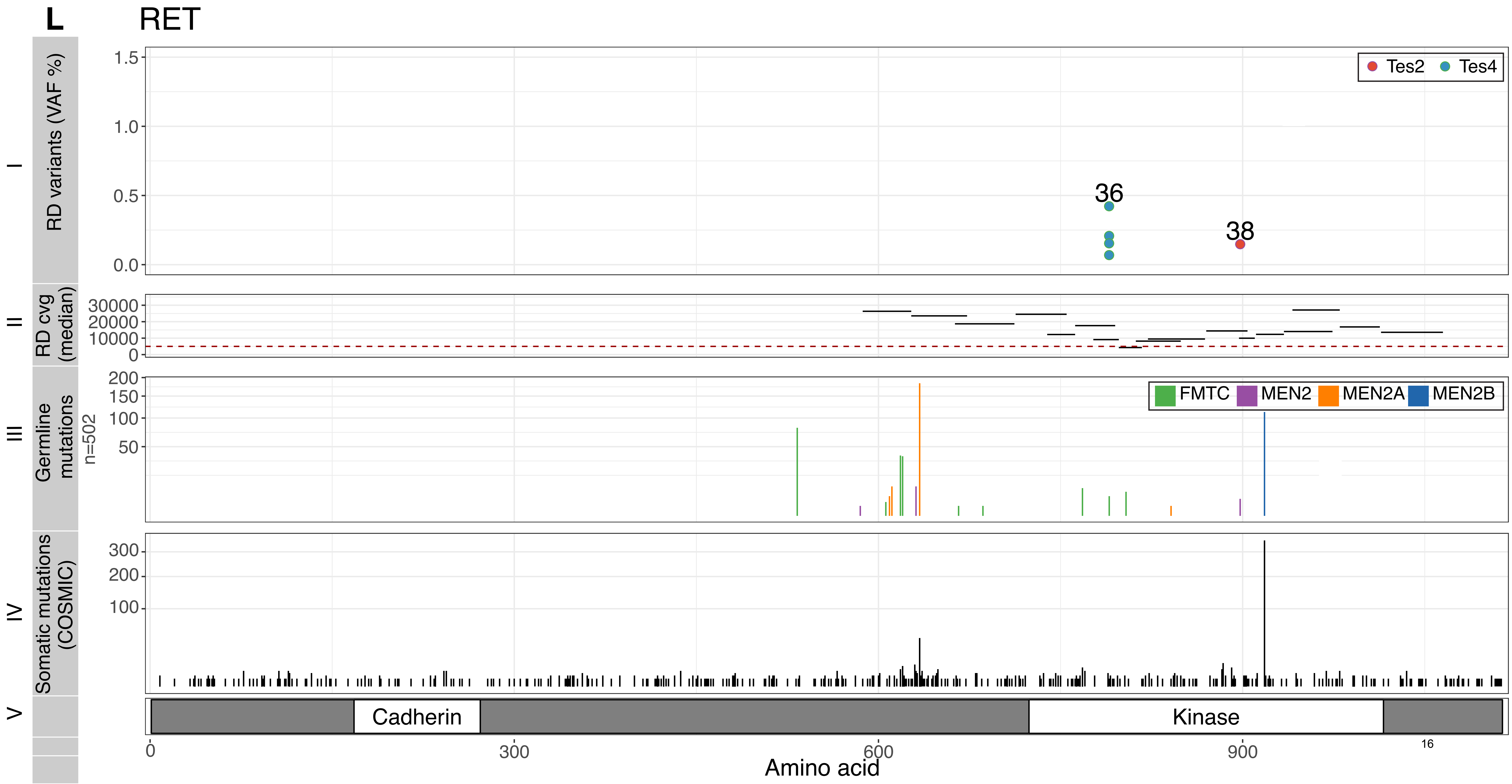

L
RET
I
RD variants (VAF %)
1.5
1.0
0.5
0.0
Tes2
Tes4
36
38
II
RD cvg (median)
30000
20000
10000
0
III
Germline mutations
n=502
200
150
100
50
FMTC
MEN2
MEN2A
MEN2B
IV
Somatic mutations (COSMIC)
300
200
100
V
Cadherin
Kinase
0
300
600
900
Amino acid

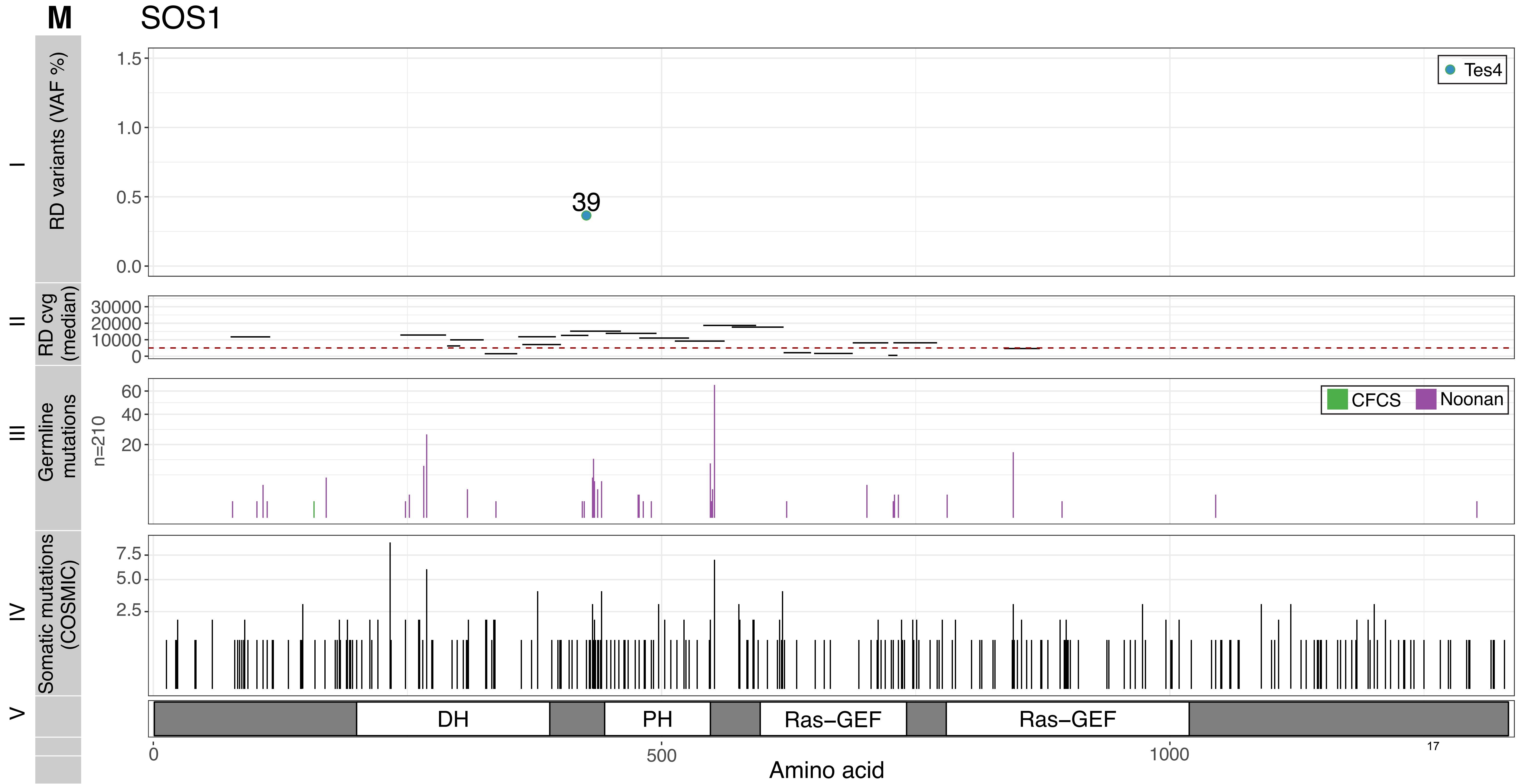
M
SOS1
I
RD variants (VAF %)
1.5
1.0
0.5
0.0
Tes4
39
II
RD cvg (median)
30000
20000
10000
0
III
Germline mutations
n=210
60
40
20
CFCS
Noonan
IV
Somatic mutations (COSMIC)
7.5
5.0
2.5
V
DH
PH
Ras−GEF
Ras−GEF
0
500
1000
Amino acid

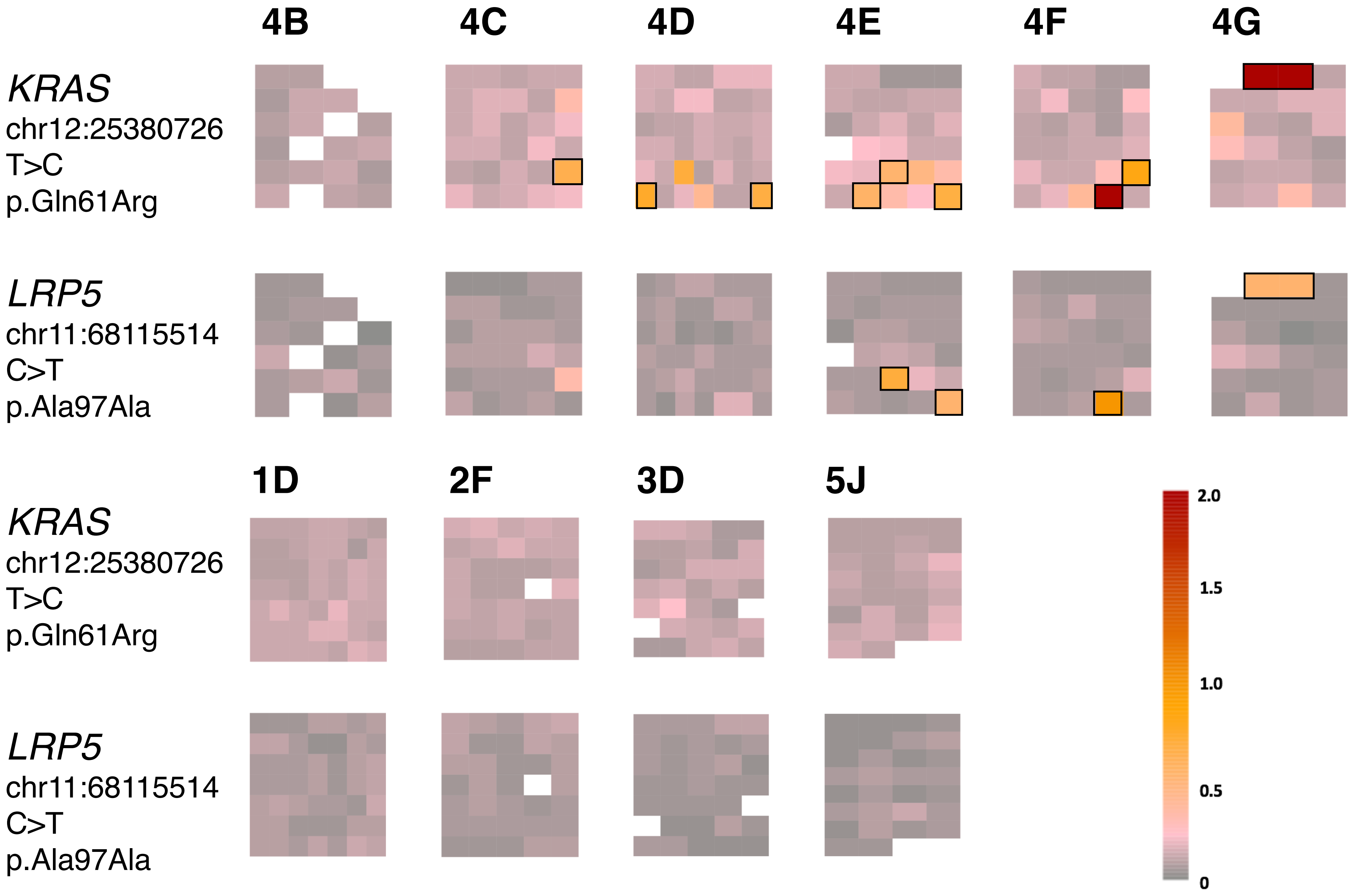

Supplemental Fig 4: Variant allele frequencies of *KRAS* c.182A>G (p.Gln61Arg) and *LRP5* c.291C>T (p.Ala97=) in all 276 biopsy samples.

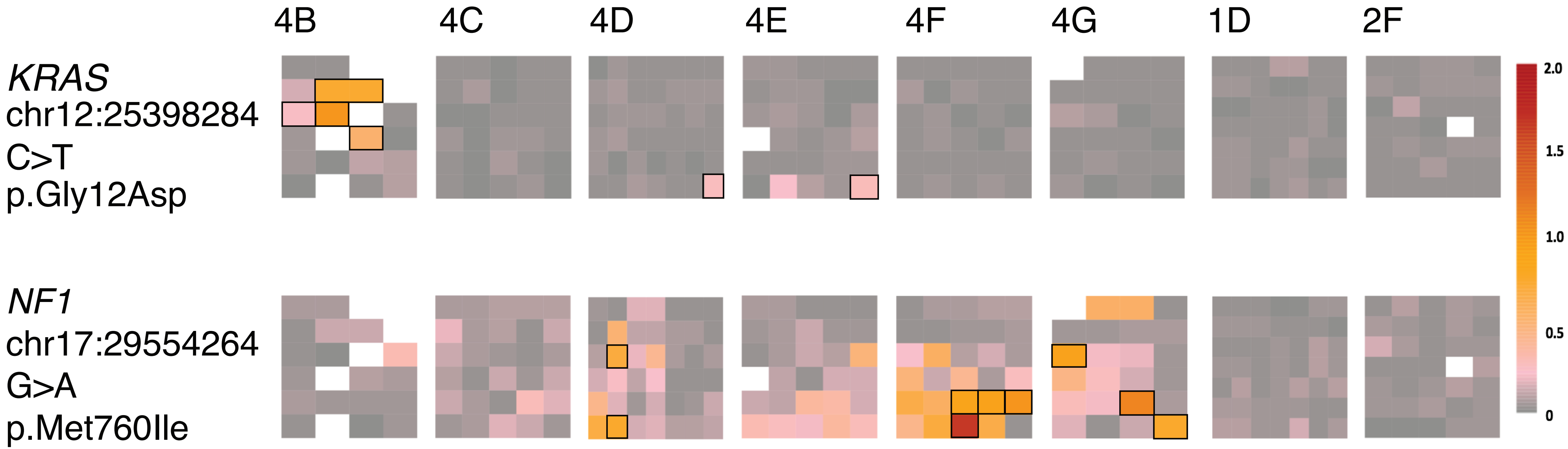

Supplemental Fig S5: Heatmap of *NF1* c.2280G>A and *KRAS* c.35G>A. Heatmap of G>A variants in *NF1* (called in 9 biopsies in Tes4 – surrounded by black lines) and *KRAS* (called in 6 biopsies in Tes4 – surrounded by black lines) reveals that there were a number of additional biopsy samples with relatively high levels of the *NF1* c.2280G>A variant that were not called in our pipeline. Heatmaps for the same variants in Tes1 and Tes2 demonstrate that the higher levels are specific to Tes4.

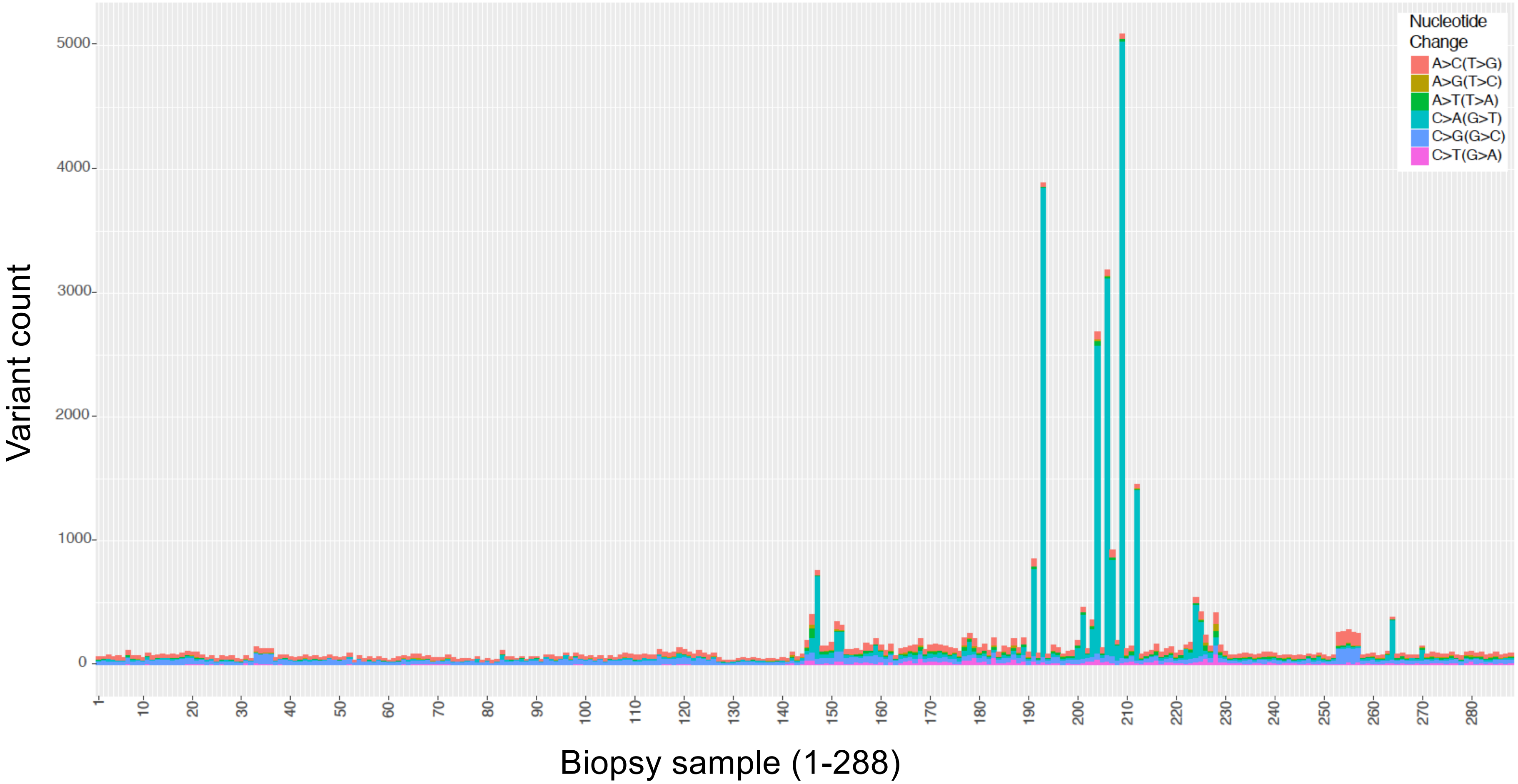

Supplemental Fig S6 – Number of variants called in each sample, organized by substitution type.
A number of biopsy samples showed an excessive load of C>A (=G>T) substitutions, a mutational signature associated with oxidative stress, likely having occurred during the experimental procedure. Filtering of specific biopsy sample-substitution types combinations and/or amplicons with excessive number of variants resulted in a dataset of 6054 variant calls.

Gene	Codon	Selfish Mutation	Germline disorder	COSMIC v82	Sperm data references	Testis biopsy data references	Immunohistochemical identification in testis & sequencing of tubule cross-sections reference
FGFR2	p.Ser252^	c.755C>G (p.Ser252Trp)	Apert syndrome	Yes	Goriely et al., 2003^; Goriely et al., 2005^; Qin et al., 2007; Choi et al., 2008; Yoon et al., [illegible]	Qin et al., 2007; Choi et al., 2008; Choi et al., 2012	-
FGFR2	p.Ser252^	c.755C>T (p.Ser252Leu)	Crouzon/Pfeiffer syndrome	-	Goriely et al., 2003^; Goriely et al., 2005^	-	-
FGFR2	p.Pro253	c.758C>G (p.Pro253Arg)	Apert syndrome	Yes	Yoon et al., 2007	Choi et al., 2008	Maher et al., 2016
FGFR2	p.Trp290	c.870G>T (p.Trp290Cys)	Pfeiffer syndrome	Yes	-	-	Maher et al., 2016
FGFR2	p.Tyr340	c.1019A>G (p.Tyr340Cys)	Pfeiffer syndrome	-	-	-	Maher et al., 2016
FGFR2	p.Cys342	c.1024T>A (p.Cys342Ser)	Crouzon/Pfeiffer syndrome	-	-	-	Maher et al., 2016
FGFR3	p.Arg248	c.742C>T (p.Arg248Cys)	Thanatophoric dysplasia I	Yes	-	-	Maher et al., 2016
FGFR3	p.Tyr373	c.1118A>G (p.Tyr373Cys)	Thanatophoric dysplasia I	Yes	-	-	Maher et al., 2016
FGFR3	p.Gly380	c.1138G>A (p.Gly380Arg)	Achondroplasia	Yes	Tiemann-Boege et al., 2002	Dakouane Giudicelli et al., 2008; Shinde et al., 2013	-
FGFR3	p.Lys650 $	multiple variants at c.1948A, c.1949A, c.1950G including p.Lys650Glu; p.Lys650Thr; p.Lys650Met; p.Lys650Asn; p.Lys650Gln	Thanatophoric dysplasia II; Familial acanthosis nigricans; Severe achondroplasia with developmental delay and acanthosis nigricans (SADDAN); [illegible]	Yes; Yes; Yes; Yes; Yes	Goriely et al., 2009	-	Maher et al., 2016 (c.1948A>G; p.Lys650Glu only)
HRAS	p.Gly12 #	multiple variants at c.34G, c.35G including p.Gly12Ser; p.Gly12Asp; p.Gly12Cys; p.Gly12Val	Costello syndrome	Yes; Yes; Yes; Yes	Giannoulatou et al., 2013	-	-
HRAS	p.Gly13	c.37G>C (p.Gly13Arg)	-	Yes	-	-	Maher et al., 2016
KRAS	p.Gln61	c.182A>G (p.Gln61Arg)	-	Yes	-	-	Maher et al., 2016
PTPN11	p.Asp61	c.181G>T (p.Asp61Tyr)	-	Yes	-	-	Maher et al., 2016
PTPN11	p.Ala72	c.215C>T (p.Ala72Val)	-	Yes	-	-	Maher et al., 2016
PTPN11	p.Asn308	c.922A>G (p.Asn308Asp)	Noonan syndrome	Yes	-	Yoon et al., 2013; Eboreime et al., 2016	-
RET	p.Met918	c.2753T>C (p.Met918Tyr)	Multiple endocrine neoplasia 2B (MEN2B)	Yes	-	Choi et al., 2012	-

^ In these studies, all possible nucleotide changes at positions c.752-755 were assessed but elevated levels were only observed for c.755C>G and c.755C>T
$ All possible nucleotide changes at positions c.1947-1952 were assessed and elevated levels were found for specific substitutions at positions c.1948-1950 only
# Most nucleotide changes (with the exception of c.35G>C (p.Gly12A)) at positions c.32-33 (p.Ala11) and c.34-35 (p.Gly12) were assessed but elevated levels were only observed for specific mutations at c.34-35

Supplemental Table S1: Literature review showing loci with evidence for selfish selection

Supplemental Table S7: Details of the different gene categories on the targeted panel (for the 431 amplicons that passed QC)

**Gene Category (see Sup Table S3)**	**RAS-MAPK**	**TEST**	**NEUTRAL-TEST**	**non-RAS-MAPK total**	**TOTAL**
Total sequenced genomic positions (bp)	25567	28345	5484	33829	59396
Total unique callable genomic positions (bp)	21599	24706	5243	29949	51548
Proportion of the panel	41.9%	47.9%	10.2%	58.1%	
Genomic positions covered by overlapping amplicons (bp)	3968	3639	241	3880	7848
% of bp in overlapping amplicons	15.5%	12.8%	4.4%	11.5%	13.2%
Number of variants in the final validated dataset	58	3	0	3	61

Supplemental Table S8: Regression coefficients describing the relative impact of several factors/variables as predictors of validated variants (ordered by *P* values).

Variable	Coefficient	Standard Error	Z value	*P*
RAS-MAPK	1.69	0.216	7.8	$6.36x10^{-15}$
Mutability	-0.784	0.122	-6.45	$1.13x10^{-10}$
Library 5	1.05	0.291	3.59	0.000327
Library 2	-1.03	0.29	-3.56	0.000372
Library 6	0.872	0.265	3.3	0.000976
Library 14	0.802	0.267	3	0.00266
Individual 3	-1.88	0.843	-2.23	0.0255
Library 13	0.763	0.373	2.05	0.0407
Library 3	0.582	0.296	1.97	0.0493
Individual 4	-1.48	0.767	-1.93	0.0538
Individual 5	-1.14	0.61	-1.87	0.0609
Individual 1	-1.44	0.782	-1.84	0.0658
Library 9	1.09	0.612	1.78	0.0743
Library 1	0.465	0.265	1.75	0.0799
Library 11	1.03	0.604	1.71	0.0865
Library 8	-0.888	0.609	-1.46	0.145
Individual 2	1.21	0.843	1.44	0.151
Library 7	-0.508	0.416	-1.22	0.222
Library 12	0.521	0.458	1.14	0.255
Library 4	0.296	0.265	1.12	0.264
Library 16	0.268	0.366	0.734	0.463
Library 10	-0.153	0.487	-0.314	0.754
Library 15	-0.062	0.311	-0.199	0.842

Supplemental Note

1. Further comments on some long-listed variants

Despite being designed to target exonic sequences, our screening panel also comprised ~18% of intronic sequences. As shown in Supplemental Table S3, five intronic variants were long-listed among the 115 different variants that passed our prioritization criteria (Supplemental Fig S1). These include a Tier 1 variant (*MAP2K2* chr19:4117383C>G), three Tier 3 variants (*KRAS* chr12:25368532A>C; *TP63* chr3:189582237A>C); *MDH1* chr2:63816228T>G) and a Tier 4 variant (*BRAF* chr7:140482986A>C). The Tier 1 and Tier 3 variants were re-screened and shown to be false positives.

Moreover, ~10% of our screening panel (Supplemental Table S7) was designed to target regions in 'neutral-test' (negative control) genes that were not anticipated to be subject to selfish selection (i.e. none of these 10 genes are considered to be cancer genes and if known as disease genes, have been associated with recessive and/or familial disorders). As shown on Supplemental Table S3, a total of 9 variants in the 'neutral-test' set were long-listed following our filtering strategy, the majority of which were in Tier 4. The only two Tier 2 variants in the neutral-test set (*GAPDH*, chr12:6646840A>G and *RHO* chr3:129251230T>C) were further re-screened by direct PCR amplification and deep-sequencing and shown to be false positive calls.

Hence among the 'neutral-test' gene set and intronic sequences, no variants validated upon re-screening, providing further support that our prioritization pipeline is able to differentiate true positive variants from technical artefacts.

2. Further comments on the *AKT3* (#1), *APC* (#2), *LRP5* (#47) variants

Among the 61 validated variants, 3 variants (*AKT3* (#1), *APC* (#2), *LRP5* (#47)) do not belong to the RAS-MAPK gene category:

Unlike the 59 other variants, the LRP5 and AKT3 variants encode synonymous substitutions that are likely to be functionally neutral. The *LRP5* c.291C>T (p.Ala97Ala) variant was present at relatively high VAF (0.53-1.20%) but as this change was called in four biopsies in Tes4 that were also positive for the driver *KRAS* c.182A>G variant (p.Gln61Arg - oncogenic) (Supplemental Figs S2 and S4), we suggest that it may represent a passenger mutation tracking the *KRAS* clone. Finally, the synonymous AKT3 (p.Ser472Ser) variant which occurs at a CpG dinucleotide has previously been reported in multiple populations (gnomAD, MAF = 0.049%, including 1.1% in African population) is likely to be neutral. Hence, like LRP5, this substitution that was identified in a single biopsy which also carried 2 other selfish variants (PTPN11/SHP2 p.Phe71Leu and BRAF p.Gln709Lys) may represent a passenger call tracking a selfish event.

While the *APC* c.4471T>G (p.Phe1491Val) variant which was identified in a single biopsy at a VAF of 0.47% may be functional (CADD score = 26.2), its significance in the absence of other variants in this gene remains unclear. Hence, although dominant germline mutations in this tumour suppressor, which controls ß-catenin turnover and affect the Wnt pathway, account for ~85% of cases of familial adenomatous polyposis (FAP), a cancer predisposition syndrome, this isolated result will warrant further investigation.

Supplemental Methods

Testis dissection

Ethical approval was given for the use of human testicular tissue by the Oxfordshire Research Ethics Committee A (C03.076: Receptor tyrosine kinases and germ cell development: detection of mutations in normal testis, testicular tumors and sperm). Testes with no known phenotypic indicators from five men aged 34, 71, 83, 87 and 90 years were either commercially sourced or obtained locally from research banks or post-mortems, with appropriate consent (sample details in Supplemental Table S5). Each testis was cut into slices ~3-5 mm thick and either stored frozen at -80°C or formalin-fixed. After thawing slices of frozen testis, extraneous tissue (epididymis or tunica albuginea) was removed and slices were further dissected into 21-36 biopsies (Supplemental Table S5). Biopsies were pulverized using a pestle and DNA extraction was performed using the Qiagen DNeasy Blood & Tissue Kit. Samples with insufficient DNA quantity (determined using Qubit fluorometer (Life Technologies)) or quality (determined using Nanodrop spectrophotometer (Thermo Scientific)) were excluded, resulting in a total of 276 biopsies [Tes1D (34 biopsies), Tes2F (30 biopsies), Tes3D (32 biopsies), Tes4B-4G (153 biopsies from 6 slices), Tes5J (27 biopsies)].

RainDance library preparation and sequencing

Primer pairs (tailed with common RainDance sequences (RD)) targeting 500 genomic regions (20-169 bp [average 133 bp, median 143 bp]) in 71 genes (66.5 kb in total) were designed by RainDance Technologies. The panel comprised mutational hotspots in the six established PAE genes, genes encoding other RTKs and members of the RAS-MAPK signaling pathway, genes in other pathways associated with spontaneous disorders that display narrow mutational spectra suggestive of gain-of-function effects but lacking epidemiological data for paternal

age-effect, oncogenes commonly mutated in cancer, some of which are also associated with germline disorders. The panel also comprises 50 amplicons that were designed to exonic regions of 10 genes encoding enzymes or components of large structural units. None of these are considered to be cancer genes and if known as disease genes, have been associated with recessive and/or familial disorders. Hence they represent a negative control/'neutral-test' genes set (*COX15, HMBS, MIP, GAPDH, RPL13A, MDH1, BFSP1, BFSP2, RHO, CHIC1*). Details of all targeted regions and primers used for amplification are provided in Supplemental Table S6. To maximize the number of different molecules amplified, massively parallel simplex PCR was performed using the RainDance Thunderstorm target enrichment system following the manufacturer's instructions. Briefly, for each sample, 6 µg of genomic DNA (gDNA) was sheared to an average size of 3,000 bp (using a Covaris blue AFA miniTUBE) and purified using a minElute column (Qiagen). One microliter (out of 20 µl) was run on a gel to verify that the gDNA had been sheared to the correct size range and the remaining gDNA was quantified using a Qubit fluorometer (Life Technologies). The custom primer library, 1.75 µg of sheared gDNA and PCR mix (Platinum Taq Polymerase High Fidelity reagents (Invitrogen), 2.5 mM $MgSO_4$, 0.35 µM dNTPs, 0.6 M betaine, 7% dimethyl sulfoxide (DMSO), in 25 µl volume) were loaded onto a ThunderStorm enrichment chip (48 samples at a time). Droplets containing up to 5 primer pairs were merged with gDNA droplets to generate an average of 2×10^6 droplets per sample (525,000 haploid genomes; average of 1 haploid genome per 3-4 droplets; on average ~1000 genomes were amplified per individual primer pair (Supplemental Fig S1). Given that for each sample, the average material input was ~1000 haploid genomes, the detection limit of the assay is anticipated to be ~0.1%. Hence, at very low VAFs (<0.1%), such as those observed for the validated Tier 2 *BRAF* (#40) call (VAF = 0.06%, supported by 14/23,601 reads), all mutant reads may have originated from a single progenitor

molecule/droplet reaction and therefore VAFs may not be entirely accurate. Following the merge, libraries were PCR-amplified (94°C for 2 min; 54 cycles of 94°C, 54°C, 68°C for 30 s each; 68°C for 10 min) and the emulsion was broken down with 75-100 µl of Droplet Destabilizer (RainDance) before being purified using AMPure beads (Agencourt). An aliquot of each sample was run on a Bioanalyzer high sensitivity chip (Agilent) to verify the amplification profile and determine the sample concentration. Sixteen different Illumina sequencing tailed libraries were constructed using a set of of 18 different barcoded (8 bp barcode (BC)) Illumina PE2-RD-rev adaptors (BC1-BC18), a common PE1-RD-Fwd, 4 ng of merged amplicons and Phusion Hot Start Flex DNA Polymerase (New England BioLabs) with 8% DMSO (98°C for 30 s, followed by 10 cycles of 98°C for 15 s, 56°C for 30 s, 72°C for 40 s, and a final extension at 72°C for 10 min). Following purification (Qiagen MinElute), the relative concentration of the secondary tailing PCR samples was estimated by Real-Time PCR using PE1 and PE2 primers. For each of the 16 libraries, 18 samples with BC1-18 were pooled in equimolar ratio and each final library was diluted to 10 nM. A total of 288 samples (264 singletons and 12 in duplicate) were amplified across 6 ThunderStorm enrichment chips (48 samples each) and subsequently ultra-deep sequenced (~22,000×) on two flow cells (16 lanes; 18 samples per lane) of Illumina HiSeq 2000 (2 × 100 bp) using RD-Read1 and RD-Read2 custom sequencing primers generating 14-20 × 10^7 paired-end reads per library. All primer sequences are given in Supplemental Table S6.

Sequence alignment, variant calling and prioritization

Low quality reads with more than 20 bases below Q20, read pairs with one or two short (<50 bp) reads and reads pairs with unmatched or mismatched sequences between the forward and reverse primer pairs expected for each amplicon were removed. Reads passing QC (on

average 86% of reads) were aligned to the human genome (hg19) using BWA-MEM version 0.7.10 (Li 2013) with default parameter settings. Primer sequences were included in the alignment but ignored during variant quantification. The Python library Pysam (https://github.com/pysam-developers/pysam) was used to fetch reads mapped to each amplicon and mapped bases (indicated as letter "M") were identified from the CIGAR string. Pileup was then performed for each amplicon independently. Nine amplicons that did not map to the targeted genomic regions were excluded from subsequent analyses (Supplemental Table S2). After trimming, reads with more than 10 non-reference bases were removed (<1% of coverage on average). For amplicons shorter than 200 bp, to avoid double-counting reads at positions where Read 1 and Read 2 overlapped, only the base with the higher quality was considered. At each position, the consensus (reference) allele was determined as the allele with the highest read count.

Data exploration of the non-consensus variant counts within each amplicon across the different samples revealed clear data structure with differences between flow cells, sequencing lanes, coverage depths and base quality scores. To reduce false positive calls, primer sequences were trimmed and only variants supported by at least 10 reads were called. To account for the technical confounders, the data was normalized (accounting for flow cell, lane, and average base quality at each position) using a simple linear model

$$y_{i,s} = f_s + l_s + n_s + q_{i,s} + \epsilon_{i,s}$$

where $y_{i,s}$ is the nucleotide count for biopsy sample s at position i; f_s, l_s and n_s are the flow cell identifier, the sequencing lane identifier, and individual identifier for biopsy sample s respectively; and $q_{i,s}$ is the average base quality of sample s at position i. We used the glm function in R for model inference (glm(y ~ f + l + n + q, family=gaussian())). Values of $\epsilon_{i,s}$

represent the normalized signals after accounting for the technical confounders and were used as inputs for the subsequent analyses. To further account for the effect of the sequencing lane structure, we removed the median effect from each lane to reduce the background noise. Let $m_{i,l} = median_{s\in l}(\epsilon_{i,s})$ be the median value at site *i* for lane *l*, computed from all biopsy samples in a sequencing lane. The adjusted quantification is $y_{i,s} = \epsilon_{i,s} - m_{i,l_s}$, where l_s is the lane for biopsy sample s. We further stabilize the variance using the transformation $\tilde{y}_{i,s} = y_{i,s}/IQR(y_i)$, where IQR($y_i$) is the inter-quantile range at site *i* across all biopsy samples. Following these normalization steps, variant calling was performed using a normal model to test for an increase in non-consensus allele frequencies.

Under the null hypothesis of no increase in VAF, we assume the normalised quantification follows a normal distribution:

$$y_i \sim N(\hat{\mu}_i, \hat{\sigma}_i)$$

(also indicated on the figure key) where $\widehat{\mu_i}$, and $\hat{\sigma}_i$ are the mean and variance at *i* estimated from all biopsy samples. We then test for elevation of VAF in the biopsy samples with the following hypothesis

$$H0 : y_{i,s} \leq \widehat{\mu_i}$$

$$H1 : y_{i,s} > \widehat{\mu_i}$$

which can be done in R (pnorm(y, mean=mu, sd=sigma, lower.tail=FALSE)).

Each non-reference nucleotide (i.e. allele, ACGT) at each genomic position across the 288 samples was tested independently in each amplicon that passed QC. Variant prioritization was performed using a P-value cutoff of $-log_{10}P > 20$, which resulted in a total of 19,625 genomic positions with at least one non-reference call. Details of the codes can be found in the Supplemental Custom pipeline or online at https://github.com/zd1/raindance))

Biopsy samples or amplicons with an excessive number of variants were more likely to represent technical artifacts. Hence, for each sample, at each site where a non-reference call was made, we used a Chi-squared test to test for excessive mutation load (-log10(P) > 3), considering each of the possible substitution types (i.e. A>C; A>G; A>T; C>A; C>G; C>T), with the null expectation being the median of each substitution type. Seven amplicons and 185 specific biopsy sample-substitution type combinations were removed from further analysis. Notably, the majority of these were C>A (=G>T) variant calls (Supplemental Fig S6), which represent a known mutational signature associated with oxidative stress that likely arose during sample preparation (Arbeithuber et al. 2016; Chen et al. 2017). Further filtering was performed to remove potential sources of artifacts: calls positioned 1 base from the amplification primer's 3'-end were excluded; calls with a maximum VAF of ≥3% were excluded to avoid calling inherited SNPs and to eliminate gross alignment errors or calling of non-consensus variants resulting from homologous genomic regions or pseudogene amplification; positions with a median depth coverage across all samples below 5,000× were excluded (this removed a 53 further amplicons (10.6%) from the analysis; Supplemental Table S2. This resulted in a total of 5729 calls (or 5659 distinct variants across 431 amplicons (51.5 kb) in 67 genes) at 5421 positions passed these filters, the majority (90.2%) of which were made in a single amplicon and sample.

As singleton calls were more likely to represent PCR or sequencing artifacts, we further prioritized calls made in two or more samples and/or present in overlapping amplicons. To exclude potential batch effects, variants were excluded if all calls were made from a single library and the number of calls was >3. This strategy identified 374 variants at 361 genomic positions. VAFs across all samples at each of the 361 genomic positions were plotted and manually inspected for sequencing library preparation or batch effects; raw sequencing reads

from calls with suspected sequence misalignment were visualized in Integrative Genomics Viewer (IGV) (Robinson et al. 2011). Variant calls showing evidence of library-specific batch or sequence misalignment effects were excluded from further analysis. Variants in *PTPN11* that matched bases at homologous positions in one of its four pseudogenes were also excluded. The remaining 115 variants at 105 genomic positions were annotated with ANNOVAR version 2015Jun17 (Wang et al. 2010). Full details of the 115 variants are presented in Supplemental Table S3. If a variant was covered by more than one amplicon, or was present in a replicated biopsy, the VAFs presented in Table 1 and the Figures represent the mean allele frequency of the called variants. Supplemental Fig S1 summarises the experimental design and the main data processing steps.

Variant validation

DNA from at least one putative-positive biopsy sample and at least 8 control samples (unrelated blood gDNA and gDNA from other testicular biopsies) was screened by PCR amplification or by single molecule molecular inversion probes (smMIPs) capture and ultra-deep sequencing (~30,000×) using Illumina MiSeq 300v2 (PCR) or 150v3 (smMIP) kits (primer and smMIP details in Supplemental Table S6). For PCR, 60 ng of gDNA was amplified (30 cycles with Phusion High-Fidelity polymerase (NEB)) in duplicate using sequence-specific primers (distinct from those used for Raindance amplification) tailed with generic common sequence 1 (CS1) or CS2 (Fluidigm). Diluted PCR products were indexed for Illumina sequencing by PCR (8 – 10 cycles with iProof high-fidelity polymerase (Bio-Rad)) using Access Array Barcode Library primers (Fluidigm). smMIPs capturing target regions were designed using the MIPgen algorithm (Boyle et al. 2014). Pools of smMIPs were phosphorylated using T4 Polynucleotide Kinase (NEB) (0.4 U per µl of 100 µM smMIPs) at 37°C for 45 min, followed by heat inactivation

at 65°C for 20 min. 100 ng of sample DNA was incubated with each MIP pool, at a 1600:1 molar ratio of smMIPs:DNA. Following denaturation at 95°C for 10 min, samples with incubated for 24 hr at 60°C with 3.2 U polymerase (Hemo Klentaq (NEB)) and 1 U ligase (Ampligase (Epicentre)). Unbound smMIPs and template DNA were removed by incubating with 1 U exonuclease I (NEB) and 5 U exonuclease III (NEB) for 45 min, followed by heat inactivation at 95°C for 2 min. Circularized smMIPs with captured regions were split into eight aliquots, each of which was amplified and barcoded by PCR (22 cycles with iProof high-fidelity polymerase) using primers targeting consensus sequences on the smMIP backbone (Supplemental Table S3). Barcoded PCR and smMIP products were purified with AxyPrep magnetic beads (Axygen).

Demultiplexed reads were aligned to the human genome (hg19) using BWA-MEM version 0.7.12 (Li 2013). Summary tables of the calls across the aligned target region for PCR and were generated using SAMtools mpileup. A base call was only considered if its mapping quality was ≥Q20 and phred score ≥Q30. Pileups for smMIP-sequenced samples were obtained using a custom pipeline: UMIs were extracted from reads and grouped using UMI-tools (Smith et al. 2017). Reads were further trimmed to remove primers, assigned to the probe they were amplified from, and aligned to GRCh37 (without alt contigs) using bwa mem version 0.7.12 (Li 2013). Pileup tables, containing the number of read pairs supporting each base call at each position were then generated using custom scripts written in Python 3.5.3 with pysam (https://github.com/pysam-developers/pysam), biopython (Cock et al. 2009) and pandas (McKinney 2010). Reads were filtered to remove low-quality (MAPQ < 20) and flagged alignments. Furthermore, base calls from UMI groups not supported by at least 2 reads, where no majority (>50%) of reads agreed on the consensus base call, or where the highest

observed base call quality was less than Q30 were also removed. This entire pipeline, built using Snakemake version 3.11.2 (Koster and Rahmann 2012), will be available from https://github.com/koelling/amplimap/. Validated variants were annotated according to the following transcripts - *APC*: NM_001127510, *AKT3*: NM_005465, *BRAF*: NM_004333, *CBL*: NM_005188, *FGFR2*: NM_000141, *FGFR3*: NM_000142, *KRAS*: NM_033360, *LRP5:* NM_002335, *MAP2K1*: NM_002755, *MAP2K2*: NM_030662, *NF1*: NM_001042492, *PTPN11*: NM_002834, *RAF1*: NM_002880, *RET*: NM_020975, *SOS1*: NM_005633.

Immunohistochemistry, microdissection and targeted mutation screen

Where mutations had been identified in frozen sections for which an adjacent FFPE tissue block was available, we attempted to visualize the corresponding mutant clone in sections of the FFPE block. Immunohistochemical staining with anti-MAGEA4 antibody (clone 57B, gifted by Prof. Giulio C. Spagnoli) to identify tubules with enhanced spermatogonial MAGEA4 staining, followed by laser capture microdissection and DNA extraction of adjacent FFPE sections, was performed as described (Maher et al. 2016). DNA was subsequently amplified by PCR (40 cycles) using CS-tagged primers (Supplemental Table S6) and barcoded for Illumina MiSeq 300v2 sequencing as described above. DNA samples extracted from the whole tissue section and from adjacent tubules with a normal MAGEA4 staining appearance were used as controls. Reads were aligned to the human genome (hg19) using BWA-MEM version 0.7.12 (Li 2013) and were visualized in IGV.

Analysis of variant enrichment

In order to test for enrichment of variants in the RTK-RAS-MAPK pathway, we performed a Fisher's exact test, categorizing each genomic position tested as to whether it was part of a

gene in the RTK-RAS-MAPK pathway category (or not), and whether we found a validated variant at the genomic position in the final validated dataset or not (Supplemental Table S7). To further evaluate the contribution of different variables to this observed enrichment, we conducted a logistic regression analysis. As predictors we used whether a variant belongs to a gene in the RAS-MAPK pathway (or not), the individual testis donors identity (ind), the sequencing libraries (lib 1-16), and types of substitutions (Mutability). The response was whether the variant was part of the final validated dataset or not.

$$logit(p(\text{ validated})) = \text{RAS-MAPK} + \text{mutability} + \text{ind1} + \ldots + \text{ind5} + \text{lib1} + \ldots + \text{lib16}$$

where ind1-5 and lib1-16 indicate the five individual testes and the sixteen libraries. The model was fitted using the glm function in R.

The measurements of the individual and sequencing library variables are obtained by summing the non-consensus read counts (NCRs) across individuals and libraries respectively. The Mutability is obtained by summing the NCRs across the entire data set. We observe significant positive coefficient for the RAS-MAPK pathway variable ($P = 6.36 \times 10^{-15}$), suggesting that the significant enrichment observed previously with the Fisher's exact test is not an artifact of mutability or sequencing libraries/individual effects. The regression coefficients are shown in Supplemental Table S8.

References

Arbeithuber B, Makova KD, Tiemann-Boege I. 2016. Artifactual mutations resulting from DNA lesions limit detection levels in ultrasensitive sequencing applications. *DNA Res* **23**: 547-559.

Boyle EA, O'Roak BJ, Martin BK, Kumar A, Shendure J. 2014. MIPgen: optimized modeling and design of molecular inversion probes for targeted resequencing. *Bioinformatics* **30**: 2670-2672.

Chen L, Liu P, Evans TC, Jr., Ettwiller LM. 2017. DNA damage is a pervasive cause of sequencing errors, directly confounding variant identification. *Science* **355**: 752-756.

Cock PJ, Antao T, Chang JT, Chapman BA, Cox CJ, Dalke A, Friedberg I, Hamelryck T, Kauff F, Wilczynski B et al. 2009. Biopython: freely available Python tools for computational molecular biology and bioinformatics. *Bioinformatics* **25**: 1422-1423.

Koster J, Rahmann S. 2012. Snakemake--a scalable bioinformatics workflow engine. *Bioinformatics* **28**: 2520-2522.

Li H. 2013. Aligning sequence reads, clone sequences and assembly contigs with BWA-MEM. *arXiv* doi:arXiv:1303.3997v2.

Maher GJ, McGowan SJ, Giannoulatou E, Verrill C, Goriely A, Wilkie AO. 2016. Visualizing the origins of selfish de novo mutations in individual seminiferous tubules of human testes. *Proc Natl Acad Sci U S A* **113**: 2454-2459.

McKinney W. 2010. Data Structures for Statistical Computing in Python. In *SciPy conference*.

Robinson JT, Thorvaldsdottir H, Winckler W, Guttman M, Lander ES, Getz G, Mesirov JP. 2011. Integrative genomics viewer. *Nat Biotechnol* **29**: 24-26.

Smith T, Heger A, Sudbery I. 2017. UMI-tools: modeling sequencing errors in Unique Molecular Identifiers to improve quantification accuracy. *Genome Res* **27**: 491-499.

Wang K, Li M, Hakonarson H. 2010. ANNOVAR: Functional annotation of genetic variants from next-generation sequencing data. *Nucleic Acids Res* **38**.
